# Quantifying Quality of Reaching Movements Longitudinally Post-Stroke: A Systematic Review

**DOI:** 10.1177/15459683211062890

**Published:** 2022-01-31

**Authors:** M. Saes, M. I. Mohamed Refai, B. J. F. van Beijnum, J. B. J. Bussmann, E. P. Jansma, P. H. Veltink, J. H. Buurke, E. E. H. van Wegen, C. G. M. Meskers, J. W. Krakauer, G. Kwakkel

**Affiliations:** 1 Department of Rehabilitation Medicine, 1209 Amsterdam UMC, Vrije Universiteit Amsterdam, Amsterdam Movement Sciences, Amsterdam Neuroscience, Amsterdam, Netherlands; 2 Department of Biomedical Signals & Systems, Technical Medical Centre, 214825 University of Twente, Enschede, Netherlands; 3 Department of Rehabilitation Medicine, Erasmus MC, 6993 University Medical Centre Rotterdam, Rotterdam, Netherlands; 4 Medical Library, 1190 Vrije Universiteit Amsterdam, Amsterdam, Netherlands; 5 Department of Epidemiology and Biostatistics, Amsterdam Public Health Research Institute, Amsterdam UMC, Location VUmcAmsterdam, The Netherlands; 6 Rehabilitation Technology, Roessingh Research and Development, Enschede, Netherlands; 7 Department of Physical Therapy and Human Movement Sciences, Feinberg School of Medicine, 12244 Northwestern University, Chicago, Il, USA; 8 Departments of Neurology, Neuroscience and Physical Medicine and Rehabilitation, 1466 Johns Hopkins University, Baltimore, MD, United States; 9 Department of Neurorehabilitation, 522567 Amsterdam Rehabilitation Research Centre, Amsterdam, Netherlands

**Keywords:** systematic review, stroke, upper extremity, recovery, biomechanics, reaching, kinematics, behavioral restitution, compensation, longitudinal studies

## Abstract

**Background:**

Disambiguation of behavioral restitution from compensation is important to better understand recovery of upper limb motor control post-stroke and subsequently design better interventions. Measuring quality of movement (QoM) during standardized performance assays and functional tasks using kinematic and kinetic metrics potentially allows for this disambiguation.

**Objectives:**

To identify longitudinal studies that used kinematic and/or kinetic metrics to investigate post-stroke recovery of reaching and assess whether these studies distinguish behavioral restitution from compensation.

**Methods:**

A systematic literature search was conducted using the databases PubMed, Embase, Scopus, and Wiley/Cochrane Library up to July 1st, 2020. Studies were identified if they performed longitudinal kinematic and/or kinetic measurements during reaching, starting within the first 6 months post-stroke.

**Results:**

Thirty-two longitudinal studies were identified, which reported a total of forty-six different kinematic metrics. Although the majority investigated improvements in kinetics or kinematics to quantify recovery of QoM, none of these studies explicitly addressed the distinction between behavioral restitution and compensation. One study obtained kinematic metrics for both performance assays and a functional task.

**Conclusions:**

Despite the growing number of kinematic and kinetic studies on post-stroke recovery, longitudinal studies that explicitly seek to delineate between behavioral restitution and compensation are still lacking in the literature. To rectify this situation, future studies should measure kinematics and/or kinetics during performance assays to isolate restitution and during a standardized functional task to determine the contributions of restitution and compensation.

## Introduction

About 80% of stroke survivors suffer from upper extremity motor impairment^1^ which affects activities of daily living.^2^ Therefore, being able to use the arm to complete functional tasks is among the top ten priorities for stroke survivors, caregivers and health care professionals.^3^ Upper extremity motor impairment after stroke is comprised of weakness, diminished dexterity and abnormal muscle synergies.^4^

Most patients exhibit some degree of spontaneous recovery of upper extremity motor impairment, with 80-90% of clinical improvements occurring within the first 8-10 weeks post-stroke.^5-7^ Studies suggest that reaching movements tend to converge toward healthy patterns, without necessarily returning fully to pre-stroke patterns (ie, partial behavioral restitution).^8-10^ The ability to use the upper limb during functional tasks may further improve through the use of compensatory strategies, in which patients accomplish a functional goal in a different way than pre-stroke (ie, behavioral compensation).^11^ The ability to distinguish between behavioral restitution and compensation would help to better identify interventions that can influence true neurological recovery.

Quality of movement (QoM) reflects the degree of motor control.^12^ Despite consensus on a standardized set of clinical measures in stroke studies,^13^ these clinical measures lack the ability to capture small changes in QoM^12,14^ and cannot distinguish behavioral restitution from compensation. Longitudinal kinematic studies early after stroke are needed to investigate the time course of QoM of the upper limb. Recommendations on suitable study designs were provided by the Stroke Recovery and Rehabilitation Roundtable (SRRR) task force.^12^ The arguments in the body of the paper of the SRRR, which are implicit in the recommendations, suggest kinematic and/or kinetic measurements during 4 standardized performance assays for quantifying behavioral restitution in addition to a functional task to distinguish true recovery from compensation strategies.^12^ Performance assays are needed to quantify the different components of motor impairment: weakness, diminished finger individuation and abnormal muscle synergies. Thereby, performance assays were suggested to serve as a proxy for behavioral restitution.^12^ To capture these components of impairment, the SRRR defined the following performance assays: grip strength,^15,16^ precision grip,^16^ finger individuation,^17,18^ and 2D planar reaching.^19,20^ It was recommended to perform these measurements repeatedly in the first 6 months post-stroke. Moreover, given the nonlinear time course of recovery, these measurements should be repeated more frequently in the first months post-stroke, preferably at fixed times.^13^ Investigating these performance assays is not only important to quantify behavioral restitution the in absence of compensation, the association between performance assays and clinical assessments may also elucidate which motor impairment component is most strongly represented by a clinical assessment score. This may make clear whether, for example, the Fugl-Meyer motor assessment of the upper extremity (FM-UE), a clinical assessment commonly used in stroke rehabilitation, truly captures synergy-driven intra-limb coupling or to which degree it is contaminated by other motor impairment components such as strength.^21,22^ Furthermore, to determine the degree to which recovery has converged on normal movement, the SRRR recommended that a healthy control group should be included.^13^ A recent review showed that the number of studies that use kinematics and kinetics to investigate reaching performance is growing exponentially.^23^ However, the focus of that particular review was not on longitudinal studies, nor on the metrics that distinguish between behavioral restitution and compensation.

Our objective was to review the literature on the use of kinematic and/or kinetic metrics to measure recovery of QoM after stroke. We focused on upper limb reaching and pointing tasks, as they require coordination of the elbow and shoulder, which is an important component of many daily activities and is often limited post-stroke as a result of weakness, loss of motor control and the intrusion of abnormal muscle synergies.^19,24^ We aimed to:

(1) identify longitudinal studies that used kinematic and/or kinetic metrics reflecting QoM to investigate post-stroke recovery of reaching, to show the reported responsiveness of these metrics over time, and their longitudinal association with clinical measures and
(2) assess whether these studies have addressed or provided suggestions on how to best capture behavioral restitution and distinguish it from compensation during a reaching task.

## Methods

### Search Strategy

A systematic literature search was performed based on the Preferred Reporting Items for Systematic Reviews and Meta-Analysis (PRISMA) statement^25^ and registered in PROSPERO (number CRD42018100648). To identify all relevant publications, systematic searches were conducted (by MS, MIMR, and EJ) in the databases PubMed, Embase, Scopus (Elsevier) and the Cochrane Library (Wiley) from inception to July 1st, 2020. Search terms included controlled terms from MeSH in PubMed and Emtree in Embase as well as free text terms. Free text terms only were used in Scopus and the Cochrane Library. Search terms expressing “stroke” were used in combination with search terms comprising “reach and grasp activity” and “kinematics and kinetics.” Search filters for human studies and English language were used. Reference tracking was performed to identify other relevant publications. Finally, duplicate articles were removed. The full search strategies for all databases can be found in Supplementary Material.

### Study Selection

After the initial literature search, the titles and abstracts of all papers found were screened independently by 2 researchers (MS and MIMR). Differences of opinion were discussed, and if no consensus was reached a third reviewer (EW) was approached. Criteria for inclusion were (1) adult participants who suffered from a stroke; (2) use of a repeated measures study design with at least 2 serial within-subject measurements starting before the chronic phase (<6 months)^11^ post-stroke; and (3) at least 1 kinetic or kinematic outcome metric, measured with any device that does not interfere (ie, disturb/restrict) with the specific movements assayed during an active goal-oriented reaching or pointing task. A study was excluded when (1) it was a review or conference proceeding; or (2) the investigated population consisted of fewer than ten subjects; or (3) it was not written in English. Investigated cohorts were allowed to be part of an intervention study. A full-text version of all remaining studies was obtained for thorough reviewing by the researchers (MIMR and MS) to establish the definitive inclusion.

### Data Analysis

#### Definitions

Behavioral restitution was defined as changes of movement execution patterns that made them more similar to those observed in healthy subjects.^11^ Behavioral compensation was defined as regaining the ability to accomplish a goal through substitution with a new movement approach that differs from pre-stroke behavior.^11^ Performance assays were defined as tests that quantify aspects of affected motor control performance in the absence of compensatory movements and outside the context of a functional task.^12^ Quality of movement was defined as a measure of patient’s motor task execution in comparison with age-matched normative values of healthy individuals.^12^ An extensive list of definitions of other terms can be found in Supplemental Material.

#### Data Extraction

The following data were extracted (when applicable): (1) authors and date of publication; (2) sample size; (3) characteristics of included participants; (4) assessment moments; (5) authors’ description of the investigated reaching task; (6) the performed clinical sensory and motor assessments; (7) measurement setup (equipment, segments, sample frequency, dimensions, and number of repetitions); (8) definitions of the investigated kinematic and kinetic metrics; (9) the change of the outcome metrics over time; (10) association of metrics with clinical assessments; (11) psychometric properties (validity, reliability, and responsiveness) of these metrics; and (12) investigated performance assays.

#### Data Interpretation

First, in the Longitudinally Investigated Kinematic and Kinetic Metrics section, an overview is provided regarding the reported metrics, how they are used to quantify movement trajectories, their responsiveness (ie, change over time) and longitudinal association with clinical measures.

Thereafter, in the Metrics Reflecting Behavioral Restitution or Compensation Strategies section, we described any suggestions made by the authors of the studies on how to track behavioral restitution or distinguish restitution from compensation. We discussed what the reviewed studies reported about kinematics in association with behavioral restitution and/or compensation. We also assessed whether the study design of the articles is compatible with recent recommendations of the SRRR for studying QoM post-stroke using kinematics and/or kinetics.^12,13^ This was only meant as a retrospective review, as most of the studies included in this review were conducted before the task force’s recommendations were published. The SRRR recommendations concern measurement time points and measurement methods, such as (1) performing the first measurement within or before the early subacute phase (≤3 months) post-stroke, when changes in QoM are still to be expected due to spontaneous neurobiological recovery; (2) inclusion ≤1 week post-stroke, pursuing an inception cohort; (3) perform measurements at fixed time points post-stroke^5,6^; (4) repeat measurements at least in weeks 1, 12, and 26 post-stroke; (5) presence of reference data of age-matched nondisabled subjects; (6) use high-resolution digital optoelectronic systems to capture movements; (7) use a sample frequency ≥60 Hz; (8) ≥ 15 movement repetitions; and (9) investigate performance assays related to motor impairments^12^ in addition to the reaching task.

## Results

### Study Identification

**Figure 1.**
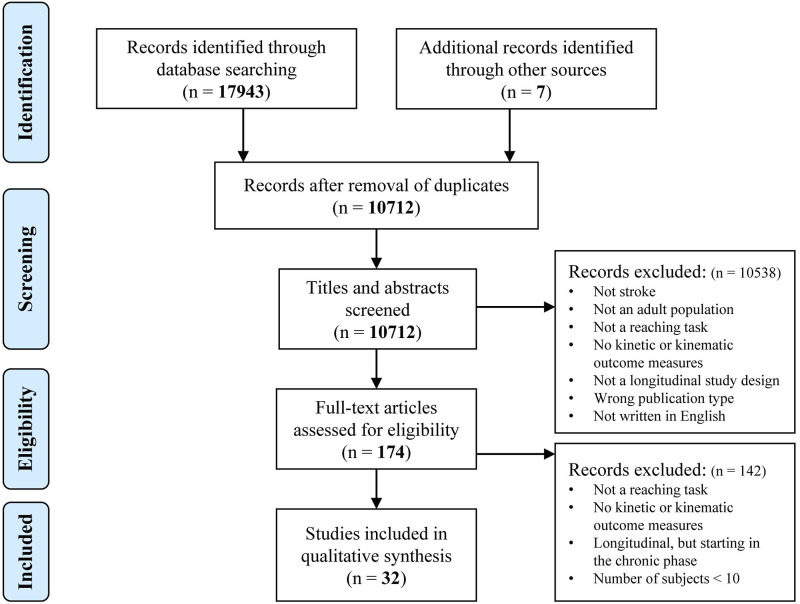
PRISMA flow diagram of included studies.

The PRISMA flow diagram of the search and selection process is presented in Figure 1. The literature search generated a total of 17943 references: 6063 in PubMed, 6678 in EMBASE, 1839 in Scopus, and 3363 in The Cochrane Library. After removing duplicates, 10712 references remained. Of these articles 10538 were discarded after reviewing title and abstract. The full-text of the remaining 174 articles was assessed for eligibility.^26^ Thirty-two articles, involving a total of 1259 unique patients with a hemorrhagic or ischemic stroke, met all criteria and were included in the current systematic review.^8-10,27-55^ Table 1 shows detailed characteristics of the included studies.

**Table 1.** Characteristics of Included Studies.

Authors	Objective	Study Type and Number of Subjects	Participants	Stroke	Assessment Moments	Reaching Task; Postural Restrictions	Additional Performance Assays Investigated	Clinical Measures	Protocol	Equipment
*Authors; cohort*	*Objective of the article*	*Study type; subject type (*N*)*	*Age: Mean (SD); gender: (male/female)*	Inclusion in days post-stroke (SD); type (I/H); affected side (L/R or *D/ND)	*Mean* ± *SD in days/weeks/months*	*Task as described by the authors; trunk compensation restricted (yes/no)*	*To identify behavioral restitution in absence of compensation*	*Clinical tests and neurophysiological techniques*	*Dimensions (1D/2D/3D); number of repetitions; sample frequency*	*System; segments*
Platz et al, 2001; NR	Test the efficacy of the arm ability training on a sample of patients with central arm paresis after traumatic brain injury or stroke	Interventional; S_AAT_ (S:16, TBI:4 S_AAT+KR_ (S:14, TBI:6 S_no AAT_ (S:15, TBI:5)	49 (17.9); 11/9 54 (18.0); 14/6 58 (15.3); 11/9	42.7 (25.2); NR; 12/8 43.4 (49.7); NR; 14/6 72.1 (139.3); NR; 11/9	Pre and post 3 weeks of intervention	Horizontal forward reach of 200 mm, start and end on table; no	x	TEMPA	2D; 20; 100 Hz	Tablet; Stylus
Rohrer et al, 2002; NR	To provide additional evidence to literature that recovery proceeds by progressive blending of sub-movements, by quantifying the smoothness of movements made by stroke patients and how it changed over the course of recovery	Interventional; S_subacute_ (12) S_chronic_ (19)	55.7 (13.7), 56.2 (17.3); 21/10	30; NR; NR 914.2 (45.7); NR; NR	Pre- and post-intervention	Point to point reaching; no	x	FM-UE	2D; as many as possible; NR	MIT Manus; Ha
Lang et al 2006a, VECTORS^1^	Examine the responsiveness and validity of the Action Research Arm Test (ARAT) in a population of subjects with mild-to-moderate hemiparesis within the first few months after stroke	Interventional; S (51)	63.7 (13.6); 21/29	9.5 (4.5); 39/11; *21/29	9.5 ± 4.5 d PS, 25.9 ± 10.6 d PS, 110.8 ± 20.7 d PS	Move the hand from thigh to the target at 90% of arm’s length in front of the affected shoulder; yes	x	ARAT, NIHSS, FIM, sensory assessment	3D; 3; 60 Hz	6-Camera’s; Tr, UE, Fa, dorsum of Ha, Th, IF, T
Lang et al, 2006b; VECTORS^1^	Examine the relative recovery of reach vs grasp from the acute to chronic phase following stroke	Interventional; S (23) C (10)	64.5 (12.8); 12/11 59.1 (12.5); 5/5	9.1 (3.5); 10/5; *9/14	9.1± 3.5 d PS, 105.3±18.8 d PS, 383.4 ±16.3 d PS	Move the hand from thigh to the target at 90% of arm’s length in front of the affected shoulder; yes	x	ARAT, sensory assessment, MAS, muscle strength	3D; 3; 60 Hz	6-Camera’s; Tr, UE, Fa, dorsum of Ha, Th, IF, T
Wagner et al, 2007; VECTORS^1^	How do sensorimotor impairments relate to reaching performance in the subacute phase after stroke and how do sensorimotor impairments measured in the acute phase after stroke relate to reaching performance measured several months later	Interventional; S (39) C (10)	63.9 (11.5); 15/24 59.1 (12.5); 5/5	8.7 (3.6); 25/8; 18/21	8.7 ± 3.6 d PS, 108.7 ± 16.5 d PS	Forward reaching from thigh to 90% of arm’s length at shoulder height in front of affected shoulder; yes	Individuation indexes (shoulder, elbow, and wrist). Max grip strength, Jamar handheld dynamometer	ARAT, FM-UE, sensory assessment, MAS, AROM	3D; 3; 60 Hz	6-Camera’s; Tr, UE, Fa, dorsum of Ha, Th, IF, T
Konczak et al, 2010; NR	How is lesion site and arm dysfunction associated in the acute stage and what is the course of upper limb recovery during the first 4 months	Observational; S (16)C (10)	60.1 (14.4); 11/5 59.0 (10.3); 7/4	14.5 range 1-33; 16/0; 7/9	14.5d PS (range 1–33), 2w after session 1, 3m after session 1	1. Point at a ball suspended from the ceiling in front at 90% of arm’s length at shoulder height. 2. Point at the same location in absence of the target; no	x	MCS, MRI	3D; 10; 100 Hz	3D ultrasound-based motion analysis system; finger
Dipietro et al, 2012	Investigate whether untrained and trained movements were characterized by similar changes in smoothness and sub-movements	Interventional; S_subacute_ (42) S_chronic_ (116)	61.3 (1.8); 24/18 58.8 (1.2); 73/43	19.1 (1.2); NR; 32/10 1150 (90); NR; 53/63	Pre-, halfway and post-intervention	Eight targets, surrounding a center target, were displayed on a monitor Subjects moved from the center 14 cm to each target, stopped, then returned to the center; No	x	FM-UE	2D; 80; NR	MIT-Manus and its commercial version InMotion2; Ha
Edwards et al, 2012; VECTORS^1^	Examine the internal consistency, validity, responsiveness, and advantages of the WMFT and compare these results to the ARAT in participants with mild-to-moderate hemiparesis within the first few months after stroke	Interventional; S (51)	63.7 (13.6); 21/29	9.5 (4.5); 39/11; 21/29	9.5 ± 4.5 d PS, 25.9 ± 10.6 d PS, 110.8 ± 20.7 d PS	Move the hand from thigh to the target at 90% of arm’s length in front of the affected shoulder; yes	x	WMFT, ARAT, sensorimotor impairments, FA, FIM, NIHSS.	3D; 3; 60 Hz	6-Camera’s; Tr, UE, Fa, dorsum of Ha, Th, IF, T
Tan et al, 2012; NR	Identify the effects of CIMT on anticipatory hand posture selection and movement time for task-specific reach-to-grasp performance	Interventional; S_CIMT_ (10) S_No CIMT_ (10)C (6)	59.7 (11.2); 7/3 58.2 (13.4); 5/5 43.8 (5.0); NR	228 (56); NR; 11/9.1191 (1225); NR; 4/6	Pre and post 2 weeks of intervention	2 different objects, 2 different grasp types. Grab the object and place it in the hole 15 cm from the edge of the table; yes	x	WMFT, MAL	1D; 7; NR	Electric switches at home position, object and target; NA
Colombo et al, 2013; HUMOUR	We aimed to analyze how time since the acute event may influence the motor recovery process during robot-assisted rehabilitation of the upper limb	Interventional; S_subacute_ (20) S_chronic_ (21)	58.4 (12.9); 8/12 50.7 (11.3); 14/7	69 (42); 15/5; 12/8 876 (1221); 17/4; 10/11	Pre and post 3+ weeks of intervention	The handle of the robot is grasped by the patient and moved through the workspace of the device (ie, in the horizontal plane). The task consisted of a sequence of point to point reaching movements in the shape of a geometrical figure; yes	x	FM-UE, MAS	2D; NR; 100 Hz	2 DoF elbow-shoulder manipulators MEMOS; end effector
Duret and Hutin, 2013; NR	Analyze clinical and kinematic motor outcomes during an intensive upper limb robot-assisted training program performed as an adjunct to a standard rehabilitation program over an extended period in the subacute phase after stroke in patients with moderate-to-severe paresis	Observational; S (10)	47.5 (19.6); 3/7	53.5 (15.8); 8/2; 6/4	1±1 d; 40±4 d; 80±6 d; 120±13 d PI	Reaching task toward targets set in 4 compass directions. Each movement was 14 cm; no	x	FM-UE, MSS	2D; 1-3; NR	InMotion 2.0 robot; end effector
Metrot et al 2013a NR^2^	To assess the natural evolution of reaching kinematics during standard post-stroke rehabilitation, focusing on bimanual coordination	Observational; S (12)	65.6 (9.7); 9/3	20.6 (7.1); 8/4; 5/7	Inclusion, 1w, 2w, 3w, 4w, 5w, 6w and 12w PI.	Grasp a ball on the table 25 cm away from the starting position of the hand; yes	x	FM-UE	3D; 5; 30 Hz	Electromagnetic motion tracker; Ha
van Kordelaar et al, 2013; EXPLICIT^3^	Assess longitudinal improvements in dissociated upper limb movements during a standardized reach-to-grasp task in patients with a first-ever ischemic stroke	Observational; S (31) C (12)	60.0 (11.2); 18/13 52.8 (5.9); 7/5	14 (6); 31/0; 19/12	14d, 25d, 38d, 57d, 91d and 189d PS	Move hand from edge of the table in front of affected shoulder to grasp a block at maximum reaching distance of the non-paretic arm; No	x	NIHSS, ARAT, FM-UE, BI	3D; 4; 240 Hz	Electromagnetic motion tracker; Tr, Sc, E, Wr
Krebs et al, 2014; NR	Predicting clinical outcomes with robot-assisted measurement of kinematic and kinetic with sufficient accuracy to serve as their surrogates	Observational; S_completers_ (87) S_non-completers_ (121)	69.7 (13.5); 45/42 75.7 (13.0); 61/60	7; 87/0; 44/43 7; 121/0; 67/54	7d, 14d, 21d, 30d, and 90d PS	Visually guided and visually evoked reaching and circle drawing movements, and attempts to move against resistance; No	x	NIHSS, mRS, FM-UE, MP	2D; as many as possible; NR	MIT Manus; Ha
Van Dokkum et al 2014 NR^2^	Addressing the link between clinical and kinematic assessment of motor performance during early post-stroke recovery	Observational; S (13) C (12)	63.9 (9.4); 10/3 32.5 (11.4); 0/12	21.2 (7.2); 9/4; 5/8	1w, 2w, 3w, 4w, 5w, 6w, and 3m PI	Grasping a ball on the table 20 cm in front of the patient and bring it to a target 5 cm from the edge of the table; yes	x	FM-UE	3D; 5; 30 Hz	Electromagnetic motion tracker; Ha
van Kordelaar et al, 2014; EXPLICIT^3^	Investigate the time course of recovery in terms of smoothness of upper limb movements in the first 6 months post-stroke, and assess how progress of time contributes to normalization of this metric	Observational; S (44)	58 (12); 25/19	<7(NR); 44/0; 27/17	1w, 2w, 3w, 4w, 5w, 8w, 12w, and 26w PS	Move hand from edge of the table in front of affected shoulder to grasp a block at maximum reaching distance of the non-paretic arm; no	x	NIHSS, FM-UE, ARAT, BI	3D; 7; 240 Hz	Electromagnetic motion tracker; Tr, Sc, UA, Fa, Ha, Th, IF.
Bang et al, 2015; NR	To investigate the effects of a modified constraint-induced movement therapy (mCIMT) with trunk restraint in subacute stroke patients	Interventional; S_exp_ (9) S_control_ (9)	60.2 (5.8); 5/4 59.3 (8.2); 4/5	90 (34); 6/3; 6/3 107 (30); 5/4; 4/5	Pre and post 4 weeks of intervention	Reaching forward to grasp a cube, placed in the sagittal plane in the trunk midline at mid-sternal height at arm length; yes	x	ARAT, FM-UE, BI, MAL	3D; 3; NR	Dartfish motion analysis software; S, E, Wr
Li et al, 2015; NR	Investigate the concurrent validity of kinematic variables before and after the intervention and the predictive validity after the intervention during reaching tasks with and without a trunk constraint in individuals with stroke	Interventional; S (95)	57.1 (10.9); 30/65	519(NR); NR; 42/53	Pre and post 3–4 weeks of intervention	Reach the index finger toward the bell at 90% of arm’s length; yes/no depending on condition	x	FM-UE, ARAT, MAS	3D; 3; 120 Hz	7-camera’s (VICON); Tr, Sh, UA, Fa, Wr, IF
Prange et al, 2015; Early Arm Support	To examine the effect of weight-supported arm training combined with computerized exercises on arm function and capacity, compared with dose-matched conventional reach training in subacute stroke patients	Interventional; S_exp_ (33) S_control_ (33)	60.3 (9.7); 17/18 58.0 (11.4); 24/19	51.1 (23.8); 28/7; NR 47.6 (21.7); 25/8; NR	Pre and post 6 weeks of intervention	Start with hand as close to the sternum as possible and reach forward maximally. Movement was performed in free space to prevent any support; yes	x	FM-UE, SULCS	2D; 5; NR	Arm support device (ArmeoBoom); Wr
Semrau et al, 2015; NR	Quantify proprioceptive and motor deficits using robotic technology during the first 6 months post-stroke to characterize timing and patterns in recovery, and compare robotic assessments with traditional clinical measures	Observational; S (113)	NR; NR	10.6 (6.6); NR; NR	1w, 6w, 12w, and 26w PS	8-target center-out reaching task. Each movement was 10 cm; no	x	TLT, CMSA, FIM, Purdue Pegboard	2D; NR; NR	Exoskeleton (KINARM); robot reflects position of Ha
Yoo et al, 2015; NR	Examine the effects of upper limb robot-assisted therapy in the rehabilitation of stroke patients	Interventional; S (15)	40–49:8, 50–59:3, 60 + :4; 13/2	0-6 m: 10, >7 m: 5; 11/4; 7/8	Pre and post 4 weeks of intervention	Move the hand from center position to targets in each of 8 compass directions (distance not clarified); no	x	FM-UE, MBI	2D; NR; NR	MIT MANUS; Sh, E
Buma et al, 2016; EXPLICIT^3^	Investigate the association between jerk and recruitment of secondary sensorimotor areas. Is this association different in the early subacute phase compared to the chronic phase post-stroke	Observational; S (17)	59.9 (12.6); 14/3	41 (8); 17/0; 12/5	5.9±1.1 w PS; 28.8±1.2 w PS	Move hand from edge of the table in front of affected shoulder to grasp a block at maximum reaching distance and transport it to target location at the contralateral side at reaching distance; no	x	FM-UE, ARAT, NHPT, fMRI	3D; 7; 240 Hz	Electromagnetic motion tracker; Tr, Sc, UA, Fa, Ha, Th, IF.
Duret et al, 2016; NR^4^	Investigate the relationships between clinical and kinematic motor outcomes after an upper limb robot-assisted training program added to usual care in patients with severe paresis, in the subacute phase of stroke	Interventional; S (38)	56 (17); 19/19	55 (22); 29/9; 23/15	Pre- and post-intervention (35 days in between)	Center-out point-to-point unconstrained reaching tasks without assistance toward visual targets set in 8 compass directions (14 cm apart) and presented in a clockwise order; no	x	FM-UE, MSS	2D; 80; NR	InMotion 2.0 arm robot; end effector
Cortes et al, 2017; SMARTS	To isolate and characterize the time course of recovery of arm motor control (kinematics of antigravity reaching movement) and clinical tests over the first year post-stroke	Observational; S (18) C (12)	55.0 (12.9); 9/9 58.4(NR); NR	13.13 (13.23); 18/0; *6/12	1.5w, 5w, 14w, 27w and 54w PS	Straight movement to a target arrayed radially at 80 mm from a central starting point, 8 directions; yes	x	FM-UE, ARAT, strength of m. biceps	2D; 80; 130 Hz	Kinereach apparatus with anti-gravity support and Flock of Birds; Ha
Pila et al, 2017; EudraCT Trial	Measure overall changes associated with a 3-month robot-assisted training program coupled with conventional care, on motor impairment and pointing task kinematics of the upper limb in late subacute stroke. Also, to compare the course of the various kinematic parameters over time, and the associated clinical changes at different joints	Observational; S (22) C (17)	53 (18); 13/9 53 (18); 8/9	63 (29); 15/7; 10/12	63 ± 29 d PS 98 ± 32 d PS 131 ± 28 d PS 167 ± 31 d PS	Reaching toward visual targets in 3 directions, each movement was a 14 cm horizontal hand displacement; yes	x	FM-UE	2D; >300; NR	InMotion; Ha
Palermo et al, 2018; NR	Investigate whether kinematic indices, based on motion capturing a 3D daily-life inspired gesture, improved after the administration of an RMT protocol, which involved an exoskeleton for 3D upper limb rehabilitation, and how these indices are in agreement with patient assessments that have been assessed using the most widely adopted clinical scales for post-stroke motor impairment	Interventional; S (10)	60.1 (18.3); 8/2	120 (45); NR; 5/5	Pre and post 4 weeks of intervention	Reach and point at a target, placed on the subject’s sagittal plane, at shoulder height, and at a distance from the body equal to the patient’s arm length; no	x	FIM, BI, FAT, FM-UE	3D; 6; 120 Hz	Optoelectronic System (BTS SMART-DX 300) consisting of 6 infrared CCD cameras; both arms: Fa, Ha, Wr_ulna_, Wr_radio_, E, C7, Sacrum, targets
Mazzoleni et al, 2018; NR	(i) To investigate the relationship between wrist training and proximal segment recovery; (ii) to compare the recovery of subacute and chronic stroke patients after wrist robot-assisted rehabilitation training	Interventional; S (20)	66.4 (16.2); 9/11	25.4 (16.0); 17/3; 8/12	Pre and post 6 weeks of intervention	Move the cursor from the center of the screen to each of 8 peripheral targets. Only N/E/S/W directions were used for analyses; yes	x	FM-UE, FM_Shoulder-Elbow_, FM_wrist_, MAS_wrist_, MI-UE, Box and Block test	2D; 16; NR	InMotion WRIST robot. 3 DOF (abduction-adduction, flexion-extension, pronation-supination); Wr
Duret et al, 2019; NR^4^	Examine a range of variables in order to identify reliable indictors of upper limb motor performance following an intensive rehabilitation program that combined 16 sessions of robot-assisted training (3 days/week) with usual care during the subacute phase in patients with moderate-to-severe upper limb paresis following stroke	Interventional; S (46)	57 (17); 25/21	58 (22); 32/14; 24/22	Pre and post 5 weeks of intervention	80 point-to-point reaching movements toward 8 visual targets, each 14 cm from the center position; no	x	FM-UE FM_shoulder-elbow_	2D; 80; NR	InMotion 2.0 Arm robot, with 2 active translational degrees of freedom to assist shoulder (flexion/extension) and elbow (flexion/extension) movements in the horizontal plane; Ha
Mazzoleni et al, 2019; NR	Investigate the effectiveness of combining tDCS and wrist robot-assisted rehabilitation in subacute stroke patients and whether this combination therapy would provide additional benefits in comparison with robotic therapy only	Interventional; S_exp_ (18) S_control_ (16)	67.5 (16.3), 8/12; 68.7 (15.8), 7/12	25 (7); 13/7; 9/11 25 (7); 16/3; 8/11	Pre and post 6 weeks of intervention	Move the cursor from the center of the screen to each of 8 peripheral targets. Only N/E/S/W directions were used for analyses; yes	x	FM-UE, FM_Shoulder-Elbow_, FM_wrist_, MAS_wrist_, MI-UE, Box and Block test	2D; 16; NR	InMotion WRIST robot. 3 DOF (abduction-adduction, flexion-extension, pronation-supination); Wr
Goffredo et al, 2019; NR	Analyze built-in kinematic data registered by a planar end-effector robot for assessing the time course of motor recovery and patient’s workspace exploration skills	Interventional; S (68)	65.28 (12.71); 45/23	NR; 49/19; 39/29	Sessions 1, 5, 10, 15, and 20 of robotic therapy	Point-to-point reaching movements toward a visual target and back, each target 14 cm from the center position; yes	x	BI, MI-UE	2D; 32 per target; 200 Hz	InMotion 2.0. Two DOF robotic device; Ha
Hussain et al, 2020; SALGOT^5^	Determining how the relationship between objective kinematic variables obtained from the target-to-target pointing task and self-reported manual ability varies during the first year after stroke	Observational; S (66)	65.7 (13.4); 39/27	9.54 d; 53/13; 29/37	10d, 4w, 3m, 6m, 12m PS	Reach and point at the target using the stylus; no	x	ABILHAND questionnaire, FM-UE	3D; 32; NR	Phantom Omni haptic stylus; Ha
Thrane et al, 2020; SALGOT^5^	To quantify longitudinal changes and residual deficits in movement performance and quality during the first year after stroke using kinematic analysis of drinking task	Observational; S (56) C (60)	64.0 (13.4), 35/31; 63.4 (12.6), 33/27	NR; NR; 30/22 (4 other)	3d, 10d, 4w, 3m, 6m, 12m PS	Reach and grasp the glass, lifting the glass and bringing it to the mouth, taking on sip of water, placing the glass back down on the table and return the arm to its initial position; no	x	NIHSS, FM-UE, FM_sensation_	3D; 3; 240 Hz	Motion capture system (ProRe-flex MCU240 Hz, Qualisys) with 5 optoelectronic cameras; Ha, Wr, E, Sh_L_, Sh_R_, Tr, head, top and bottom of the glass

^1-5^ Partial overlap of included patients between studies with the same number. *Abbreviations:* AAT: arm ability training, ARAT: Action Research Arm Test, AROM: Active Range of Motion, AS: arm support, C: healthy controls, CIMT: constraint-induced movement therapy, CMSA: Chedoke-McMaster Stroke Assessment, CON: conventional, d: days, D: dominant, E: elbow, Fa: forearm, FA: functional ability, FIM: Functional Independence Measure, fMRI: functional MRI, FM-UE: Fugl-Meyer motor assessment of the upper extremity, FM_sensation_: Fugl-Meyer domain for sensation, Ha: Hand, H: hemorrhagic, I: ischemic, IF: index finger, KR: knowledge of results, L: left, M: months, MAL: Motor Activity Log, MAS: Modified Ashworth Scale, (M)BI: (Modified) Barthel Index, MCS: motor control scores, MFS: Modified Frenchay Scale, MP: motor power, MRI: magnetic resonance imaging, MSS: Motor Status Scores, NA: not applicable, ND: non-dominant, NHPT: Nine Hole Peg Test, NIHSS: National Institutes of Health Stroke Scale, NR: not reported, PI: post-inclusion, PS: post-stroke, R: right, S: stroke patients, Sc: scapula, SD: standard deviation, Sh: shoulder, SIS: Stroke Impairment Scale, SULCS: Stroke Upper Limb Capacity Scale, T: target, TBI: traumatic brain injury, TEMPA: Test Evaluant les Membres superieurs de Personnes Agees, Th: thumb, TLT: Thumb Localization Test, Tr: trunk, UA: upper arm, W: weeks, Wr: wrist, WMFT: Wolf Motor Function Test.

### Longitudinally Investigated Kinematic and Kinetic Metrics

#### Kinematic metrics to quantify quality of movement

**Figure 2.**
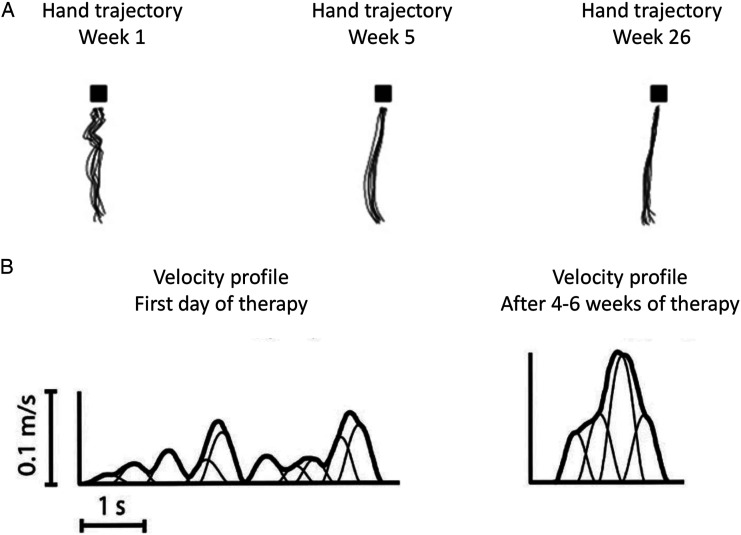
(A) (adapted from Van Kordelaar et al, 2014) Reaching trajectories of the hand of one patient in weeks 1, 5, and 26 after stroke onset. Patients move their hand from the start position to a block, in this figure visualized as a black square. Each trace represents one reach-to-grasp movement. (B) (adapted from Rohrer et al, 2004) Typical velocity profile of a stroke patient during a point-to-point movement at the first day of therapy and after 4–6 weeks of therapy.

Spontaneous neurological recovery leads to improved QoM. In healthy individuals, the movement trajectory during a standardized reaching task is close to a straight line between the starting position and the target.^19,56^ The velocity profiles of healthy individuals are smooth and bell-shaped curves with 1 clear velocity peak.^19,56^ A pre-planned and well-controlled movement results in a smooth increase of velocity whereby an adequate peak velocity is reached.^45^ Figure 2 shows 2D movement trajectories during a standardized reaching task and typical velocity profiles at different time points post-stroke.^8,57^ Through visual inspection, one can clearly conclude that QoM is affected early after stroke and improves over time, especially in the first weeks. In spite of the many metrics, there is no consensus on which metrics are best to quantify QoM and therefore behavioral restitution during functional tasks. The same applies to metrics for compensation. To address this issue, some investigators use a global measure that does not presuppose that any specific kinematic measure should be used and instead rely on the task design itself to prevent compensation.^10^ This makes a more general point that no kinematic measure can be interpreted outside of the behavioral context within which it was generated.

Figure 2 shows that in addition to visual inspection, movement trajectories can be quantified by many different kinematic metrics, each of which may be affected by different aspects of motor impairment and/or compensation. For instance, patients perform movements slower early after stroke, either due to weakness or to compensate for decreased accuracy.^58^ Early post-stroke, peak hand velocity is often decreased and the time at which this peak is reached is often delayed, reflecting slowed muscle recruitment.^45^ Movement smoothness is a widely acknowledged metric of QoM.^47,59^ Different smoothness metrics have been reported during reaching, which quantify different aspects of motor control. Metrics which have been reported include, amongst others, jerk (third derivative of hand position) and peaks metric (number of velocity peaks in the velocity profile), both have been associated with feedback corrections and the number of sub-movements.^27,47,49,57^ The deviation in movement trajectory can also be quantified by comparing the performed hand trajectory to a straight line between start position and the target (eg, path error and reach efficiency). Quality of performance in a multi-joint reaching movement can also be quantified as the accuracy in arriving at the target location (eg, endpoint accuracy), which requires adequate coordination of different joints during the movement. Besides the hand, kinematic data can be obtained from other segments of the upper extremity, which allows estimates of joint rotations (eg, elbow, shoulder, and trunk), which can also reflect either QoM or compensation.^24,60^

#### Overview of Reported Metrics

**Figure 3.**
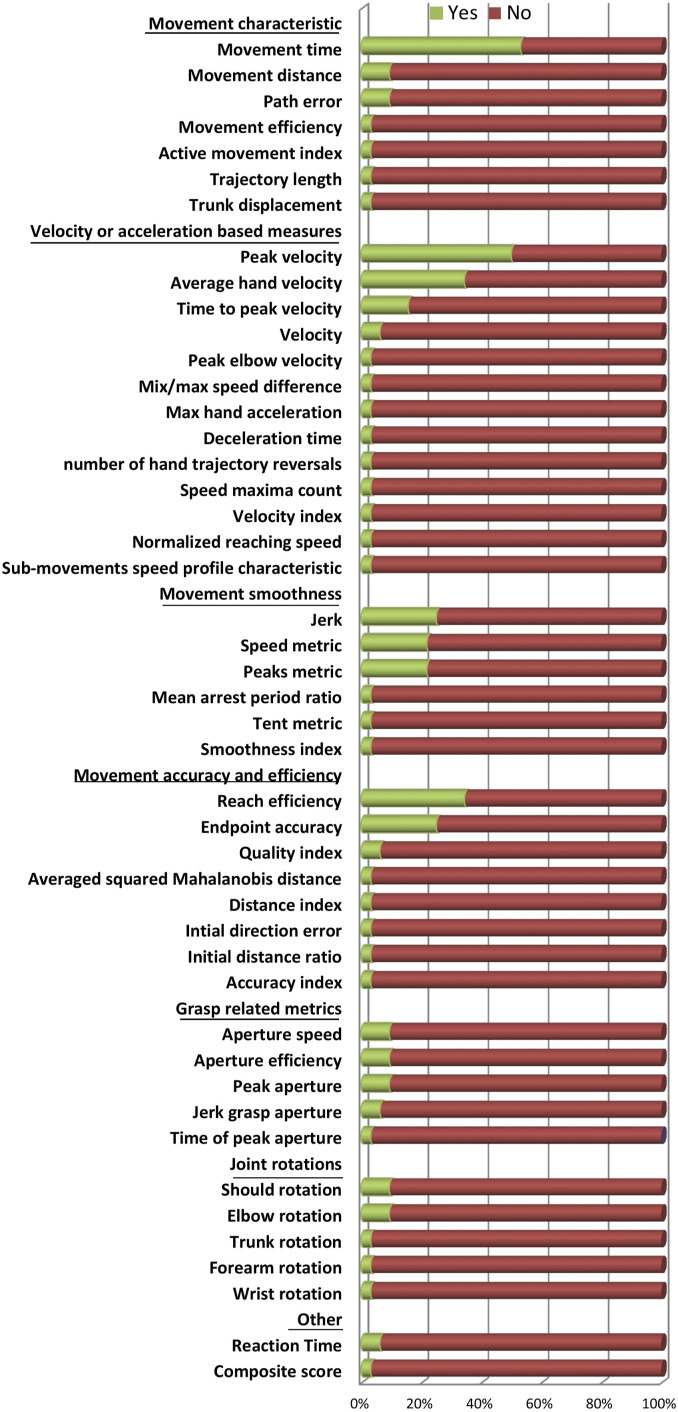
Percentage of studies which investigated a particular metric. ‘Yes’ (green) denotes the percentage of studies that included the metric in their analysis.

In total, 46 different kinematic metrics have been investigated during a reaching task in longitudinal studies starting in or before the subacute phase post-stroke (Table 2). The most frequently investigated metrics were *movement time* and *peak hand velocity* (Figure 3). Other metrics investigated in more than 20% of the studies were *average hand velocity, jerk, speed metric*, *endpoint accuracy*, and *reach efficiency*. None of the studies investigated kinetic metrics during a functional reaching task. An overview of the investigated metrics per study, including details on metric definitions as provided by the authors, and when applicable their psychometric properties, can be found in Supplementary Table.

**Table 2.** Overview of Metrics, Their Responsiveness to Change Over Time, and Their Clinical Association.

Metric in This Review	Metric Name in Study (First Author, Year)	Responsiveness Significant Change Over Time (Yes/No); Time Period Post-Stroke (T1–T2) or Passed Time (T)	Clinical Association Type: Longi/Cross (Time Point); Clinical Measure, Correlation Coefficient/NR/NS
Movement time	Movement time (Platz, 2001)	Yes; 3w	x
	Movement duration (Rohrer, 2002)	x	x
	Movement time (Lang, 2006b)	Yes; 1w–90d	x
	Movement time (Wagner, 2007)	Yes; 9d–109d	x
	Total movement time (Konczak, 2010)	Yes; 2w–4w	x
	Total movement time (Tan, 2012)	Yes; 2w	x
	Movement duration (Dipietro, 2012)	Yes; NR	x
	Movement duration (Van Kordelaar, 2013)	Yes; 14d–57d	x
	Movement time (Metrot, 2013a)	Yes; 2w, 3w	x
	Movement duration (Van Kordelaar, 2014)	Yes; 1w–5w	x
	Movement time (Van Dokkum, 2014)	x	Longi: FM-UE, *NS*
	Movement time (Semrau, 2015)	x	Cross (all): FIM, PP, CMSA, *strength NR*
	Movement time (Li, 2015)	x	Cross (pre): ARAT, FM-UE, *strength NR* Cross (post): FM-UE, *strength NR*
	Movement duration (Buma, 2016)	Yes; 6w–29w	x
	Movement time (Palermo, 2018)	Yes; 4w	Longi: FIM, BI, FAT, FM-UE; *NS*
	Task completion time (Goffredo, 2019)	Yes, NR	x
	Movement time (Hussain, 2020)	x	Cross (10 d/4 w): ABILHAND, *NS* Cross (3/6/12 m): ABILHAND, *ρ: −.46/−.49/−.75*
Movement distance	Displacement (Yoo, 2015)	No; 4w	x
	Endpoint displacement (Li, 2015)	x	Cross (pre): ARAT, *strength NR* Cross (post): ARAT, *strength NR*
	Reach distance (Prange, 2015)	Yes; 6w	x
Movement efficacy	Movement efficacy (Duret, 2013)	Yes; 40d	x
Path error	Root mean square (Duret, 2013)	No; 80d	x
	Path error (Duret, 2016)	Yes; 35d	Cross (pre): FM-UE, *ρ: −.63;* MSS, *ρ: −.63* Longi (correlation between change score): FM-UE, *ρ: −.51*; MSS, *ρ: −.49*
	Movement path error (Duret, 2019)	Yes; 5w	x
Active movement index	Active movement index (Colombo, 2013)	Yes; 3w	x
Trajectory length	Trajectory length (Van Dokkum, 2014)	x	Longi: FM-UE, *NS*
Trunk displacement	Trunk displacement (Palermo, 2018)	Yes; 4w	Longi: FIM, BI, FAT, FM-UE, *NS*
Velocity	Hand velocity (Duret, 2013)	Yes; 40d	x
	Posture speed (Semrau, 2015)	x	Cross (all): FIM, PP, CMSA; *strength NR*
Average hand velocity	Mean speed (Rohrer, 2002)	x	x
	Movement mean speed (Dipietro, 2012)	Yes; NR	x
	Mean velocity (Colombo, 2013)	Yes; 3w	x
	Average speed (Krebs, 2014)	x	x
	Mean velocity (Van Dokkum, 2014)	x	x
	Mean movement speed (Duret, 2016)	Yes; 35d	Cross (pre): FM-UE, *ρ: 0.73*; MSS, *ρ: .73* Longi (correlation between change score): FM-UE, MSS, *reported as weak*
	Mean velocity (Mazzoleni, 2018)	Yes (ab/ad component during forward and backward direction, fl/ex component during left/right direction); 6w	x
	Mean movement speed (Duret, 2019)	Yes; 5w	x
	Mean velocity (Mazzoleni, 2019)	Yes (forward, backward and left direction); 5w	x
	Movement speed (Goffredo, 2019)	Yes, NR	x
	Mean velocity (Hussain, 2020)	x	Cross (10d/4w/3m/6m): ABILHAND, *NS* Cross (12m): ABILHAND, *ρ: 0.54*
Peak velocity	Peak speed (Rohrer, 2002)	x	x
	Reach speed (Lang, 2006a)	x	*Cross (0d): ARAT, R: 0.4 *Cross (14d): ARAT, NS *Cross (90d): ARAT, *R: 0.55*
	Reach speed (Lang, 2006b)	Yes; 1w–90d	x
	Peak wrist velocity (Wagner, 2007)	Yes; 9d–109d	Cross (109 d): C-STR, *ρ: 0.55*; C-AROM, *ρ: .43*
	Max had velocity (Konczak, 2010)	Yes; 2w–4w	x
	Peak wrist velocity (Edwards, 2012)	x	*Cross (0/14/90d): WMFT function, *R: 0.63/0.35/0.45;* WMFT time, *R: −.58/NS/−.42*; WMFT grip, *R: 0.55/0.42/0.59*
	Movement peak speed (Dipietro, 2012)	Yes; NR	x
	Max reaching velocity (Metrot, 2013a)	Yes; NR	x
	Peak speed (Krebs, 2014)	x	x
	Peak hand velocity (Van Dokkum, 2014)	x	Longi: FM-UE, *NS*
	Max speed (Semrau, 2015)	x	Cross (all): FIM, PP, CMSA, *strength NR*
	Peak velocity (Li, 2015)	x	Cross (post): ARAT (significant for constrained) *strength NR*
	Peak movement speed (Duret, 2016)	Yes; 35d	Cross (pre): MSS, *ρ: 0.60*; FM-UE, *NS* Longi (correlation between change score): FM-UE, MSS, *reported as weak*
	Peak velocity (Palermo, 2018)	No	Longi: FIM, BI, FAT, FM-UE, *NS*
	Peak velocity (Hussain, 2020)	x	Cross (all): ABILHAND, *NS*
	Peak hand velocity (Thrane, 2020)	Yes; 3 d–6 m	x
Mix/max speed difference	Mix/max speed difference (Semrau, 2015)	x	Cross (all): FIM, PP, CMSA, *strength NR*
Time to peak velocity	Time of max velocity (Van Dokkum, 2014)	x	Longi: FM-UE, *NS*
	Time to peak velocity (Palermo 2018)	No	Longi: FIM, BI, FAT, FM-UE, *NS*
	Percentage of peak velocity (Li, 2015)	x	Cross (post): ARAT (significant for unconstrained); *strength NR*
	Acceleration time (Konczak, 2010)	x	x
	Relative time to peak velocity (Thrane, 2020)	Yes; 3 d–3 m	x
Max hand acceleration	Max hand acceleration (Konczak, 2010)	Yes; 2 w–4 w	x
Deceleration time	Deceleration time (Konczak, 2010)	x	x
Number of hand trajectory reversals	Number of hand trajectory reversals (Duret, 2013)	Yes; 80d	x
Speed maxima count	Speed maxima count (Semrau, 2015)	x	Cross (all): FIM, PP, CMSA, *strength NR*
Velocity index	Velocity index (Pila, 2017)	Yes; 2m–3m, 2m–4m, 2m–5m; 3m–5m	x
Normalized reaching speed	Normalized reaching speed (Mazzoleni, 2019)	Yes (abduction component during reaching in forward direction); 5w	x
Sub-movements speed profile characteristic	Number, overlap, duration, peak interval, skewness of sub-movements (Krebs, 2014)	x	x
Jerk	Jerk metric (Rohrer, 2002)	x	Longi (correlation between change scores): FM-UE, *R: −.48*
	Jerk (Dipietro, 2012)	Yes; NR	x
	Mean magnitude of jerk normalized by peak speed (Krebs, 2014)	x	x
	Root mean square of the jerk normalized by the duration of movement (Krebs, 2014)	x	x
	Normalized hand displacement jerk (Van Kordelaar, 2014)	Yes; 1w–5w	x
	Normalized jerk (Palermo, 2018)	Yes; 4w	Longi: FIM, BI, FAT, FM-UE, *NS*
	Normalized jerk (Mazzoleni, 2018)	Yes (forward and backward direction); 6w	x
	Normalized jerk (Mazzoleni, 2019)	Yes (abduction component during reaching in forward direction); 5w	x
Speed metric	Speed metric (Rohrer, 2002)	x	Longi (correlation between change scores): FM-UE, *R: 0.40*
	Speed shape (Dipietro, 2012)	Yes; NR	x
	Mean over peak speed (Krebs, 2014)	x	x
	Movement irregularity (Van Dokkum, 2014)	X	Longi: FM-UE, *NS*
	Smoothness (Yoo, 2015)	Yes; 4w	x
	Speed shape (Duret, 2016)	Yes; 35d	Cross (pre): FM-UE, *ρ: 0.75*; MSS, *ρ: 0.72* Longi (correlation between change score): FM-UE, MSS, *reported as weak*
	Smoothness (Duret, 2019)	Yes; 5w	x
Mean arrest period ratio	Mean arrest period ratio (Rohrer, 2002)	x	Longi (correlation between change scores): FM-UE, *R: 0.33*
Peaks metric	Peaks metric (Rohrer, 2002)	x	Longi (correlation between change scores): FM-UE, *NS*
	Number of peaks (Dipietro, 2012)	Yes; NR	x
	Number of velocity peaks (Metrot, 2013a)	Yes; 2w, 3w	x
	Movement smoothness (Colombo, 2013)	Yes; 3w	x
	Number of velocity peaks (Van Dokkum, 2014)	x	Longi: FM-UE, *strength NR*
	Number of peak speed (Goffredo, 2019)	Yes, NR	x
	Number of velocity peaks (Hussain, 2020)	x	Cross (10d/4w/3m/6m/12m): ABILHAND, *ρ: −.45/NS/NS/−.54/−.66*
Tent metric	Tent metric (Rohrer, 2002)	x	Longi (correlation between change scores): FM-UE, *NS*
Smoothness index	Smoothness index (Pila, 2017)	Yes; 2m–3m, 2m–4m, 2m–5m	x
Endpoint accuracy	Accuracy (Platz, 2001)	Yes; 3w	x
	Reach Accuracy (Lang, 2006a)	x	*Cross (0/14/90 d): ARAT, *R: −.53/−.50/−.45*
	Reach Accuracy (Lang, 2006b)	Yes; 1w–90d	x
	Endpoint error (Wagner, 2007)	Yes; 9d–109d	Cross (109 d): C-STR, *ρ: −.34;* C-AROM, *NS*
	Reach Accuracy (Edwards, 2012)	x	*Cross (0/14/90 d): WMFT function, *R: −.65/−.72/−.50;* WMFT time, *R: .66/0.66/0.45*; WMFT grip, *R: −.52/−.38/−.39*
	Reach error (Yoo, 2015)	Yes; 4w	x
	Reach error (Duret, 2016)	Yes; 35d	Cross (pre): FM-UE, ρ: *−*.79; MSS, ρ: *−*.79 Longi (correlation between change score): FM-UE, MSS, reported as weak
	Active range of motion (Duret, 2019)	Yes; 5w	x
Reach efficiency	Reach efficiency (Lang, 2006a)	x	*Cross (0/14/90d): ARAT, *R: −.35/−.55/−.43*
	Reach efficiency (Lang, 2006b)	Yes; 1w–90d	x
	Reach path ratio (Wagner, 2007)	Yes; 9d–109d	Cross (109d): C-AROM, *ρ: −0. 44,* Cross (109d): C-STR, *ρ: −.47*
	Reach efficiency (Edwards, 2012)	x	*Cross (0/14/90d): WMFT function, *R: −.50/−.43/−.55;* WMFT time, *R: .56/0.56/0.55*; WMFT grip, *R: −.30/−.48/−.45*
	Normalized path length (Colombo, 2013)	Yes; 3w	x
	Trajectory directness (Metrot, 2013a)	Yes; NR	x
	Deviation from straight line (Krebs, 2014)	x	x
	Trajectory directness (Van Dokkum, 2014)	x	Longi: FM-UE, *NS*
	Path length ratio (Semrau, 2015)	Yes; NR	Cross (all): FIM, PP, CMSA, *strength NR*
	Hand path ratio (Palermo, 2018)	Yes; 4w	Longi: FAT, *strength NR* (mentioned as strong); FIM, BI, FM-UE, *NS*
	Movement accuracy (Goffredo, 2019)	No	x
Averaged squared Mahalanobis distance	Averaged squared Mahalanobis distance (Cortes 2017)	Yes; 1w–5w	x
Distance Index	Distance Index (Pila, 2017)	Yes; 2m–3m, 2m–4m, 2m–5m	x
Initial direction error	Initial direction error (Semrau, 2015)	Yes; NR	Cross (1/6/12/24w): FIM, *ρ: −.61/−.56/−.47/−.52* PP, *ρ: −.79/−.73/-.72/−.77* CMSA, *ρ: −.79/−.74/−.66/−.72*
Initial distance ratio	Initial distance ratio (Semrau, 2015)	x	Cross (all): FIM, PP, CMSA, *strength NR*
Accuracy index	Accuracy index (Pila, 2017)	Yes; 2m–5 m	x
Quality index	Quality index (Mazzoleni, 2018)	Yes (forward, backward and left direction); 6w	x
	Movement error (Mazzoleni, 2019)	Yes (forward, backward and left direction); 5w	x
Aperture speed	Aperture speed (Lang, 2006a)	x	*Cross (0/14/90d): ARAT, *R: 0.58/0.35/0.39*
	Aperture speed (Lang, 2006b)	Yes; 1 w–90 d	x
	Aperture speed (Edwards, 2012)	x	*Cross (0/14/90d): WMFT function, *R: 0.65/0.40/NS;* WMFT time, *R: −.55/−.38/−.39*; WMFT grip, *R: 0.59/0.53/NS.*
Aperture efficiency	Aperture efficiency (Lang, 2006a)	x	*Cross (0/14/90d): ARAT, *R: −.45/−.6/−.45*
	Aperture efficiency (Lang, 2006b)	No; 1w–1y	x
	Aperture efficiency (Edwards, 2012)	x	*Cross (0/14/90d): WMFT function, *R: −.61/−.55/−.55;* WMFT time, *R: .52/0.55/0.50*; WMFT grip, *R: −.45/−.43/−.41*
Peak aperture	Peak aperture (Lang, 2006a)	x	*Cross (0/14/90d): ARAT, *R: .58/0.62/0.45*
	Peak aperture (Lang, 2006b)	Yes; 1w–90d	x
	Peak aperture (Edwards, 2012)	x	*Cross (0/14/90d): WMFT function, *R: 0.61/0.68/0.45;* WMFT time, *R: −.52/−.62/−.59*; WMFT grip, *R: 0.72/0.83/0.52*
Time of peak aperture	Time of peak aperture (Lang, 2006b)	No; 1w–1y	x
Jerk grasp aperture	Jerk grasp aperture (Van Kordelaar, 2014)	Yes; 1w–5w	x
	Normalized jerk grasp (Buma, 2016)	Yes; 6w–29w	Cross (w6): ARAT, *R: −.64*; FM-UE, NHPT, *NS*; fMRI, *R:[.62 .83]*
Trunk rotation	Trunk rotation (Van Kordelaar, 2013)	x	x
Shoulder rotation	Shoulder rotation (Van Kordelaar, 2013)	x	x
	Shoulder flexion (Li, 2015)	x	x
	Shoulder adduction (Li, 2015)	x	Cross (pre): FM-UE (significant for unconstrained), *strength NR* Cross (post): ARAT (significant for unconstrained), *strength NR* Cross (post): FM-UE, *strength NR*
Elbow rotation	Elbow rotation (Van Kordelaar, 2013)	x	x
	Elbow extension (Li, 2015)	x	Cross (pre): ARAT; significant for unconstrained, *strength NR*
	Maximal elbow extension (Bang, 2015)	Yes; 4w	x
Peak elbow velocity	Peak angular velocity (Thrane, 2020)	Yes; 3d–6m	x
Forearm rotation	Forearm rotation (Van Kordelaar, 2013)	x	x
Wrist rotation	Wrist rotation (Van Kordelaar, 2013)	x	x
Composite score	Composite score (Semrau, 2015)	x	Cross (all): FIM, PP, CMSA, *strength NR*
Reaction time	Reaction time (Semrau, 2015)	x	Cross (all): FIM, PP, CMSA, *strength NR*
	Reaction time (Li, 2015)	x	Cross (pre): FM-UE, *strength NR* Cross (post): FM-UE (significant for unconstrained), *strength NR*

Responsiveness was noted as change between 2 moments post-stroke or the passed time when measurement moments were not fixed post-stroke. When available, the strength of the relation was provided, R: Pearson correlation coefficient, ρ: Spearman rank correlation coefficient, *Interpreted from graph. *Abbreviations:* ABILHAND: ABILHAND questionnaire, ARAT: Action Research Arm Test, C-AROM: composite score Active Range of Motion, CMSA: Chedoke-McMaster Stroke Assessment, Cross: cross-sectional association, C-STR: composite score muscle strength, d: days post-stroke, FM-UE: Fugl-Meyer motor assessment of the upper extremity, FIM: Functional Independence Measure, Longi: longitudinal association, m: months post-stroke, MSS: Motor Status Scale, NHPT: Nine Hole Peg Test, NR: not reported, NS: not significant, Post: post-intervention, PP: Purdue Pegboard, Pre: pre-intervention, WMFT: Wolf Motor Function Test, w: weeks post-stroke, x: not investigated, y: years post-stroke.

#### Responsiveness and Longitudinal Association With Clinical Measures

Here, we report the responsiveness to change over time and the longitudinal association between kinematics and the FM-UE since this particular clinical measure was often reported by the studies. Table 2 provides an overview of responsiveness of all reported kinematic metrics to change over time and their association with clinical measures.

*Movement time, average hand velocity,* and *peak hand velocity* were shown to significantly change over time, mainly in the early (sub)acute phase post-stroke. The longitudinal association between movement time and FM-UE was not significant.^40,49^ Average hand velocity showed a poor longitudinal association with FM-UE.^38^ The longitudinal association between peak hand velocity and FM-UE was found to be weak^38^ or not significant.^40,49^ *Time to peak velocity* did not change over time,^40^ nor was it longitudinally associated with FM-UE.^40,49^

The movement smoothness metrics that were most frequently investigated in longitudinal studies after stroke were *jerk, speed metric,* and *peaks metric* (Figure 2). These metrics were shown to change over time post-stroke, mainly in the early subacute phase.^8,28,30,32,38,40,41,43^ Studies showed varying outcomes for the longitudinal association between *peaks metric* and FM-UE.^47,49^ Inconclusive results were reported for the longitudinal association between *speed metric* and FM-UE. One study showed a significant longitudinal association with FM-UE (Pearson’s r: .40),^47^ while another study found a significant but poor longitudinal association with FM-UE,^38^ and yet another study found no significant longitudinal association.^49^ Rohrer and colleagues^47^ found a significant longitudinal association between *jerk* and FM-UE (Pearson’s r: −.48), while Palermo and colleagues^40^ did not. For the smoothness metrics, *mean arrest period ratio* and *tent metric*, change over time was not investigated. *Mean arrest period ratio* was longitudinally associated with FM-UE (Pearson’s r: 0.33), while *tent metric* was not.^47^

*Endpoint accuracy* and *reach efficiency* were both responsive to change over time in the early subacute phase post-stroke. *Endpoint accuracy* was stated to be poorly longitudinally associated with FM-UE.^38^ *Reach efficiency* showed no significant longitudinal association with FM-UE.^40,49^ *Path error* was responsive to change over time and longitudinally associated with FM-UE (Spearman’s ρ: −.51).^38^

In 11 out of 32 studies, the reaching task also included grasping. In 5 of these studies, kinematic metrics for grasping were investigated.^8,37,50,51,54^ Grasp-related metrics such as *aperture speed, peak aperture* and *jerk grasp aperture* are responsive to change over time, which was not the case for *aperture efficiency* or *time of peak aperture*.^8,51^

A combination of simultaneously measured joint rotation metrics reflecting *elbow extension* and *shoulder abduction* were stated to be relevant since they are main components of stroke-related abnormal muscle synergies.^9^ In one study, a principal component analysis showed that during a reach-to-grasp task, elbow and shoulder rotations are most associated early after stroke, and become more dissociated mainly within the first 8 weeks post-stroke.^9^ In the chronic phase post-stroke, elbow and shoulder joint rotation during reaching remain more associated compared to healthy individuals.^9^ The kinematic metric *trunk displacement* is acknowledged to be a reflection of a compensation strategy to overcome the shoulder-elbow synergy that prevents elbow extension and thereby induces restriction of reaching area. The longitudinal association with clinical measures was not investigated.

### Metrics Reflecting Behavioral Restitution or Compensation Strategies

#### Attempts in the literature to investigate recovery of QoM by quantifying behavioral restitution and compensation

Trunk movement is a common compensatory strategy shown by stroke patients with any degree of motor impairment during reaching to distances that are at arm’s length.^24,61^ Trunk displacement assists the endpoint of the arm when the range of voluntary elbow extension is restricted, for example, due to affected coordination between the elbow and shoulder joints.^24^ Half of the studies intentionally restricted trunk movement during the reaching task in order to obtain kinematic data of a reaching movement which was not influenced by this form of compensation (Table 1). Three studies deliberately sought to measure compensatory movements of the trunk during a reaching task.^9,34,40^

Several studies explicitly addressed whether changes in particular metrics reflect either behavioral restitution or compensation. For example, Konczak and colleagues (2010)^53^ showed that stroke patients perform pointing movements at a slower speed compared to controls, which was independent of whether the subjects had to point in the air or at a target. From this, they concluded that moving slower is not a compensatory strategy per se. Buma and colleagues (2016)^37^ suggested that decreased movement smoothness may result from corrections of deviations from the intended optimal movement pattern. They state that jerk may reflect the control strategy to correct these deviations, which may be interpreted as a quantification of compensation.

Three studies focus on the time period in which behavioral restitution is argued to take place.^8-10^ Van Kordelaar and colleagues (2013)^9^ showed that recovery of the control over DOFs during a reach-to-grasp task, reflecting the ability to perform movements dissociated from abnormal muscle synergies,^62^ is restricted to the first 5 weeks post-stroke, while FM-UE increased until 8 weeks post-stroke. Similar findings were shown for movement smoothness.^8^ Therefore, they conclude that these kinematic metrics may quantify behavioral restitution of motor control. Cortes and colleagues (2017)^10^ investigated improvement of motor control of the upper extremity during a 2D-reaching task using the Kinereach™, which is designed to decrease strength requirements by providing antigravity support and reducing friction, while the trunk was restricted to limit compensation strategies. Thereby, the reaching task is in line with one of the performance assays suggested by the SRRR.^12^ The gravitational support does not interfere with the planar movements assayed, and allows them to be properly measured. In addition, gravity support is used to overcome shoulder weakness and thereby reduce intrusion of flexor synergies.^63^ Cortes and colleagues (2017)^10^ showed that motor control of horizontal reaching plateaued within the first 5 weeks post-stroke, whereas the FM-UE and ARAT continued to show improvements until 14 weeks post-stroke. They suggest that this difference in time window may be due to strength improvements and learning of compensatory movements contaminating the FM-UE and the ARAT, respectively. They concluded that kinematics of performance assays such as quality of 2D-reaching better isolate the underlying process of spontaneous recovery compared to clinical motor impairment scales such as FM-UE and capacity scores such as ARAT.^10^

Lang and colleagues (2006)^51^ compared recovery of reaching versus grasping after stroke. They showed that reaching accuracy recovered post-stroke, while grasping efficiency did not. It is currently unclear what the contribution of different descending pathways is concerning restitution or compensation, and what causes the difference in recovery of reaching vs grasping.

Only one study measured performance assays alongside a functional task longitudinally.^52^ Wagner and colleagues (2007)^52^ performed a reaching task and 2 performance assays: isolated joint movements and grip strength. Deficits in isolated (fractionated) movements were shown to be present by comparing the composite score of the individuation index of the shoulder, elbow and wrist to healthy controls. Also, maximal grip strength was significantly decreased in stroke patients when compared to controls. Both performance assays showed improvement over time from the acute to the subacute phase post-stroke. However, deficits in grip strength and isolated movement control remained. Normal values of kinematic metrics such as reaching accuracy and efficiency were shown during a 3D goal-directed forward reaching task, despite the remaining deficits revealed by the performance assays. On the other hand, peak wrist velocity during a reaching task remained deviated from healthy values. From this, they conclude that “*performance of functional movement can be normal or near-normal, despite the presence of underlying sensorimotor impairments. This may reflect the idea that not all functional movements require full sensorimotor capacity.*”^52^ This conclusion is in line with the present dichotomy of behavioral recovery, whereby motor function at the activity domain of the ICF is achieved by 2 components: behavioral restitution and compensation.

### SRRR Recommendation Compatibility

None of the longitudinal studies met all recommendations provided by the SRRR, one reason of course being that these recommendations were published only recently.^12^ The SRRR recommendations were predicated on the idea that it is important to distinguish between behavioral restitution and compensation. The recommendation to include longitudinal measurements of performance assays besides a functional task was met by 1 out of 32 studies. In 24 out of 32 studies, the first measurement was performed after the acute phase post-stroke, and measurements were repeated limited number of times. Furthermore, 24 out of 32 studies did not include healthy reference data and were thereby not able to determine whether observed recovery was complete. An overview of which recommendations of the SRRR were met by the individual studies is provided in Supplementary Table and Supplementary Figure. A checklist that contains all recommendations of the SRRR consensus papers is provided in Supplementary Material. This checklist can be used to design or evaluate stroke recovery studies that also target QoM by using kinematics and kinetics.

The only study which investigated recovery by performing both a functional task and performance assays^52^ met many of the recommendations of the SRRR, except for the minimal number of repetitions within a measurement, and the number of longitudinal measurements was restricted to 2 measurements per patient.

## Discussion

Despite the large number of cross-sectional kinematic post-stroke studies,^23^ longitudinal studies that track recovery of quality of upper limb movement early post-stroke remain scarce. Thirty-two longitudinal post-stroke studies were found that measured kinematic metrics during a reaching task. However, just a few of these studies addressed the need to distinguish between behavioral restitution and compensation. Only one study investigated the combination of performance assays and a functional task longitudinally,^12^ showing that metrics such as reaching accuracy and reaching efficiency normalized, while peak wrist velocity and performance assays, such as grip strength and isolated movement control, showed recovery but remained affected. This is in line with the present dichotomy of behavioral recovery, whereby performance assays reflect behavioral restitution, while the observed recovery of function in the activity domain is the sum of behavioral restitution and compensation. More longitudinal studies should investigate performance assays early after stroke in addition to functional tasks. The recommendations recently provided by the SRRR, together with the overview of reported metrics reflecting QoM, may serve as inspiration and starting point for designing stroke studies which will bring us closer to kinematics that can distinguish between behavioral restitution and compensation.

From a translational perspective, it is of interest to study the longitudinal association between the recommended performance assays and common clinical assessments. For example, in case of the FM-UE, such studies would help elucidate precisely what the measure is capturing, whether it mainly quantifies the degree to which out-of-synergy movements can be made, as was originally intended,^64,65^ or the degree to which it is contaminated by other motor impairment components, both neural and musculoskeletal.^21,22,66^ However, although some of the available studies investigated longitudinal associations between kinematics and clinical outcomes,^37,38,46,50,52,54^ these analyses did not concern kinematics obtained from performance assays.

A difference in recovery between reaching and grasping was observed by Lang and colleagues (2006).^51^ It is currently unclear what causes the difference in recovery of reaching vs grasping and what the contribution is of different descending pathways with regard to restitution and compensation. This has to be investigated by obtaining longitudinal neurophysiological data alongside kinematic data within the first months post-stroke.

Smoothness is assumed to be a good reflection of QoM. However, many different kinematic metrics have been used to quantify smoothness,^67^ which all have a different mathematical basis and therefore show varying recovery patterns. Moreover, smoothness of the hand trajectory during a reaching task can be influenced by several components of motor impairment across different joints in the upper extremity. Whether smoothness metrics are able to reflect behavioral restitution remains inconclusive and should be studied in a longitudinal study post-stroke, as recently recommended.^67,68^

In sum, this review shows that despite the growing number of cross-sectional kinematic and kinetic post-stroke studies, there is still a need for longitudinal studies that separate behavioral restitution from compensation over the course of recovery. Thus, measuring QoM remains in its infancy in stroke recovery and rehabilitation studies. Further research is necessary to provide better means to interpret neuroimaging studies^12,69,70^ and insight into which aspects of post-stroke arm function deficits are targeted during CIMT^71,72^ and neuromodulation therapies such as rTMS^73^ and tDCS.^74^ Finally, understanding recovery of QoM may aid in the design of better rehabilitation approaches targeting restitution.^12,69,75^

### Barriers in Kinematic Research Post-Stroke

There are a number of possible explanations for the paucity of longitudinal studies. First, collecting longitudinal datasets in a post-stroke cohort is challenging: having to adhere to fixed time points, at higher frequency early on; the need to restrict inclusion to those patients that can be captured in the first few weeks post-stroke; and losing patients because they often change locations during their clinical trajectory. Second, while there is agreement on QoM as proxy for true neurological recovery, and that kinematic/kinetic metrics need to be assessed,^69^ consensus on which metrics reflect behavioural restitution in absence of behavioural compensation during functional tasks is lacking. Third, there may be technology-based barriers. High-resolution optical tracking systems^12^ are typically not portable and pose a challenge for serial assessments as patients need to return to the movement laboratory for follow-up measurements, which increases the chances of drop-out. User-friendly, portable, high-resolution measurement setups or a validated setup of wearables in which inertial measurement units provide information using accelerometers and gyroscopes, would greatly improve feasibility of investigating kinematics post-stroke. An overview of the ease of application and practicality of different motion capture systems to measure kinematic metrics was recently provided.^76^ In line with the SRRR task force, authors state that markerless systems are promising for implementation in hospitals and clinics, yet require validation.^76^ Examples of such systems are the Microsoft Kinect, electromagnetic motion capture systems, and miniature inertial measurement units.^76^

### Performed Reaching Task

The performed task in the studies included in the present systematic review could either be a reach-to-grasp or reach-to-point task. It should be noted that the kinematics of these closely related tasks may differ, for example, the velocity profile. The velocity profile of a reach-to-point movement mimics a minimum jerk model,^77^ whereas the profile of a reach-to-grasp movement is more skewed.^78^ Therefore, kinematic metrics should be compared among similar tasks and no kinematic measure should be interpreted outside of the behavioral context within which it was generated. Stroke research focuses on recovery over time within subjects and comparisons with healthy subjects. This emphasizes the need for standardized tasks and the availability of reference data in healthy subjects.

The SRRR recommended to perform a functional drinking task to investigate how behavioral restitution and compensation may interact.^12^ However, only one longitudinal study included in the present review^45^ actually performed a drinking task. Studies that incorporated a drinking task were nevertheless excluded if they were either only cross-sectional^79^ or quantified the drinking task as a complete task.^80,81^ In the latter case, a global measure was obtained rather than decomposition into each kinematic phase of the drinking task (reaching, transporting glass to mouth, drinking, transporting glass to table, and returning hand). It should be noted that datasets that include a functional task like drinking might still be useful for separating restitution and compensation, if information for each separate phase can be extracted from the raw data by applying either post hoc analyses or when machine learning techniques are able to quantify quality of a complete task. A disadvantage of the drinking task is that it includes grasp, and thereby excludes patients who have very limited dexterity. Recently, an alternative task was proposed in the form of turning on/off a light switch which does not require hand function.^82^ Appropriate metrics that quantify movement quality during the light switching task are however required. Included studies investigated reaching tasks in 2D as well as in 3D. The performance assays suggested by the SRRR concern 2D movements. Currently, there are no validated 3D performance assays. Thus, currently, 3D movements, as discussed above, remain in the functional domain.

### Limitations

Due to our search restrictions regarding databases and language, some relevant studies may have been missed. Studies in which no reaching task was performed were excluded. Studies which measured performance assays but did not include a reaching task will therefore be missed. Articles often describe only part of the data obtained during the main study instead of all investigated tasks. Therefore, it might be the case that the main study meets more recommendations of the SRRR than the appraised articles. Such information can be obtained from protocol papers, which were not analyzed in this review. Finally, some of the authors (GK, EW, and JK) who contributed to the current manuscript were also part of the SRRR task force.

### Future Directions

In order to understand *what* occurs during true recovery from motor impairments after stroke and *how* innovative therapies may interact with such behavioral restitution, there is an urgent need for longitudinal studies that use kinematic and kinetic performance assays. In line with the SRRR recommendations, future studies should perform frequently repeated measurements in the first 3 months post-stroke, measurement time points should be defined as elapsed time since the moment of stroke onset and healthy reference data should be provided regarding metrics reflecting QoM. Moreover, studies targeting QoM after stroke should use different performance assays such as strength, finger individuation, reaching dexterity, and the ability to execute isolated movements for quantification of behavioral restitution. The contributions of these different motor impairment components and their relation to underlying mechanisms that drive behavioral restitution and neural repair early post-stroke need further investigation. In addition, performance assays and improvements in QoM will also allow better interpretation of observed changes in neuroimaging modalities such as EEG^83^ and fMRI^84^ obtained early post-stroke. A checklist for study design and evaluation of longitudinal kinematic/kinetic stroke studies is provided as Appendix to this manuscript (Supplementary Material).

From a technical and practical point of view, there are a number of barriers that hinder the use of high fidelity systems outside the laboratory. Therefore, we recommend the development of minimal and portable movement analysis systems or validation of existing ones to measure QoM outside the laboratory. Such portable systems will decrease patients’ burden and improve feasibility of longitudinal studies. Moreover, quick and easy to use systems are more likely to ultimately make the transition to routine clinical practice. These systems along with analysis packages that provide a small number of interpretable measures will be essential to make studying recovery using kinematics useful for clinicians.

## Supplemental Material

sj-pdf-1-nnr-10.1177_15459683211062890 – Supplemental Material for Combined Quantifying Quality of Reaching Movements Longitudinally Post-Stroke: A Systematic Review Click here for additional data file. Supplemental Material, sj-pdf-1-nnr-10.1177_15459683211062890 for Quantifying Quality of Reaching Movements Longitudinally Post-Stroke: A Systematic Review by M. Saes, M.I. Mohamed Refai, B.J.F. van Beijnum, J.B.J. Bussmann, E.P. Jansma, P.H. Veltink, J.H. Buurke, E.E.H. van Wegen, C.G.M. Meskers, J.W. Krakauer and G. Kwakkel in Neurorehabilitation and Neural Repair

sj-pdf-2-nnr-10.1177_15459683211062890 – Supplemental Material for Combined Quantifying Quality of Reaching Movements Longitudinally Post-Stroke: A Systematic Review Click here for additional data file. Supplemental Material, sj-pdf-2-nnr-10.1177_15459683211062890 for Quantifying Quality of Reaching Movements Longitudinally Post-Stroke: A Systematic Review by Mique. Saes, Mohamed Irfan Mohamed Refai, Bert-Jan F. van Beijnum, J.B.J. Bussmann, E.P. Jansma, Peter.H. Veltink, Jaap.H. Buurke, Erwin.E.H. van Wegen, Carel.G.M. Meskers, John.W. Krakauer and Gert. Kwakkel in Neurorehabilitation and Neural Repair

sj-pdf-3-nnr-10.1177_15459683211062890 – Supplemental Material for Combined Quantifying Quality of Reaching Movements Longitudinally Post-Stroke: A Systematic Review Click here for additional data file. Supplemental Material, sj-pdf-3-nnr-10.1177_15459683211062890 for Quantifying Quality of Reaching Movements Longitudinally Post-Stroke: A Systematic Review by Mique. Saes, Mohamed Irfan Mohamed Refai, Bert-Jan F. van Beijnum, J.B.J. Bussmann, E.P. Jansma, Peter.H. Veltink, Jaap.H. Buurke, Erwin.E.H. van Wegen, Carel.G.M. Meskers, John.W. Krakauer and Gert. Kwakkel in Neurorehabilitation and Neural Repair

sj-jpg-4-nnr-10.1177_15459683211062890 – Supplemental Material for Combined Quantifying Quality of Reaching Movements Longitudinally Post-Stroke: A Systematic Review Click here for additional data file. Supplemental Material, sj-jpg-4-nnr-10.1177_15459683211062890 for Quantifying Quality of Reaching Movements Longitudinally Post-Stroke: A Systematic Review by Mique. Saes, Mohamed Irfan Mohamed Refai, Bert-Jan F. van Beijnum, J.B.J. Bussmann, E.P. Jansma, Peter.H. Veltink, Jaap.H. Buurke, Erwin.E.H. van Wegen, Carel.G.M. Meskers, John.W. Krakauer and Gert. Kwakkel in Neurorehabilitation and Neural Repair

sj-pdf-5-nnr-10.1177_15459683211062890 – Supplemental Material for Combined Quantifying Quality of Reaching Movements Longitudinally Post-Stroke: A Systematic Review Click here for additional data file. Supplemental Material, sj-pdf-5-nnr-10.1177_15459683211062890 for Quantifying Quality of Reaching Movements Longitudinally Post-Stroke: A Systematic Review by Mique. Saes, Mohamed Irfan Mohamed Refai, Bert-Jan F. van Beijnum, J.B.J. Bussmann, E.P. Jansma, Peter.H. Veltink, Jaap.H. Buurke, Erwin.E.H. van Wegen, Carel.G.M. Meskers, John.W. Krakauer and Gert. Kwakkel in Neurorehabilitation and Neural Repair
