# Supplementary material for: Quantifying Quality of Reaching Movements Longitudinally Post-Stroke: A Systematic Review
Source: Neurorehabil Neural Repair. 2022 Jan 31;36(3):183–207. doi: 10.1177/15459683211062890 (PMC8902693; doi:10.1177/15459683211062890)
Supplement: sj-pdf-1-nnr-10.1177_15459683211062890 – Supplemental Material for Combined Quantifying Quality of Reaching Movements Longitudinally Post-Stroke: A Systematic Review [file sj-pdf-1-nnr-10.1177_15459683211062890.pdf]

Appendix A

Search strategy in PubMed July 1, 2020 (read from bottom-up).

Set	Search terms
#5	#4 NOT ("animals"[MeSH Terms] NOT "humans"[MeSH Terms]) Filters: English
#4	#1 AND #2 AND #3
#3	"Movement"[Mesh:NoExp] OR "Motion"[Mesh] OR "Mechanical Phenomena"[Mesh:NoExp] OR "Biomechanical Phenomena"[Mesh:NoExp] OR "Torque"[Mesh] OR "Spatio-Temporal Analysis"[Mesh] OR "Kinetics"[Mesh] OR torque*[tiab] OR biomechanic*[tiab] OR Kinematic*[tiab] OR kinetic*[tiab] OR angle*[tiab] OR force*[tiab] OR motion[tiab] OR acceler*[tiab] OR deceler*[tiab] OR rotation[tiab] OR velocity*[tiab] OR speed*[tiab] OR spatiotemporal[tiab]
#2	"Pronation"[Mesh] OR "Supination"[Mesh] OR "Hand Strength"[mesh] OR reach*[tiab] OR coordination[tiab] OR grasp*[tiab] OR grip*[tiab] OR hand strength[tiab] OR pinch strength[tiab] OR "Upper Extremity"[Mesh] OR Upper Extremit*[tiab] OR Upper Limb*[tiab] OR arm[tiab] OR arms[tiab] OR shoulder[tiab] OR elbow*[tiab] OR forearm*[tiab] OR wrist*[tiab] OR hand[tiab] OR hands[tiab] OR finger*[tiab] OR thumb*[tiab]
#1	"Stroke"[Mesh] OR "Stroke Rehabilitation"[Mesh] OR cva[tiab] OR cvas[tiab] OR poststroke*[tiab] OR post-stroke*[tiab] OR stroke*[tiab] OR apoplex*[tiab] OR cerebrovascular diseas*[tiab] OR cerebrovascular accident*[tiab] OR cerebrovascular disorder*[tiab] OR ((brain*[tiab] OR cerebr*[tiab] OR cerebell*[tiab] OR intracran*[tiab] OR intracerebral*[tiab] OR vertebrobasilar*[tiab]) AND vascular*[tiab] AND (disease[tiab] OR diseases[tiab] OR accident*[tiab] OR disorder*[tiab])) OR ((brain*[tiab] OR cerebr*[tiab] OR cerebell*[tiab] OR intracran*[tiab] OR intracerebral*[tiab] OR vertebrobasilar*[tiab]) AND (haemorrhag*[tiab] OR hemorrhag*[tiab] OR ischemi*[tiab] OR ischaemi*[tiab] OR infarct*[tiab] OR haematoma*[tiab] OR hematoma*[tiab] OR bleed*[tiab]))

Search strategy in Embase.com July 1, 2020 (read from bottom-up).

Set	Search terms
#5	#4 AND ('article'/it OR 'article in press'/it OR 'review'/it) AND [english]/lim
#4	#1 AND #2 AND #3
#3	'movement (physiology)'/de OR 'limb movement'/de OR 'arm movement'/exp OR 'hand movement'/exp OR 'motion'/de OR 'velocity'/exp OR 'mechanics'/de OR 'biomechanics'/exp OR 'force'/exp OR 'kinematics'/exp OR 'kinetics'/de OR 'torque'/exp OR 'temporal analysis'/exp OR 'spatial analysis'/de OR torque*:ti,ab OR biomechanic*:ti,ab OR kinematic*:ti,ab OR kinetic*:ti,ab OR angle*:ti,ab OR force*:ti,ab OR motion:ti,ab OR acceler*:ti,ab OR deceler*:ti,ab OR rotation:ti,ab OR velocity*:ti,ab OR speed*:ti,ab OR spatiotemporal:ti,ab
#2	'pronation'/exp OR 'supination'/exp OR 'hand strength'/exp OR reach*:ti,ab OR coordination:ti,ab OR grasp*:ti,ab OR grip*:ti,ab OR 'hand strength':ti,ab OR 'pinch strength':ti,ab OR 'upper limb'/exp OR 'upper extremit*':ti,ab OR 'upper limb*':ti,ab OR arm:ti,ab OR arms:ti,ab OR shoulder:ti,ab OR elbow*:ti,ab OR forearm*:ti,ab OR wrist*:ti,ab OR hand:ti,ab OR hands:ti,ab OR finger*:ti,ab OR thumb*:ti,ab
#1	'cerebrovascular accident'/exp OR cva:ab,ti OR cvas:ab,ti OR stroke:ab,ti OR apoplex*:ab,ti OR poststroke*:ab,ti OR ((brain*:ab,ti OR cerebr*:ab,ti OR cerebell*:ab,ti

	OR intracran*:ab,ti OR intracerebral*:ab,ti OR vertebrobasilar*:ab,ti) AND vascular*:ab,ti AND (disease:ab,ti OR diseases:ab,ti OR accident*:ab,ti OR disorder*:ab,ti)) OR (cerebrovascular*:ab,ti AND (disease:ab,ti OR diseases:ab,ti OR accident*:ab,ti OR disorder*:ab,ti)) OR ((brain*:ab,ti OR cerebr*:ab,ti OR cerebell*:ab,ti OR intracran*:ab,ti OR intracerebral*:ab,ti OR vertebrobasilar*:ab,ti) AND (haemorrhag*:ab,ti OR hemorrhag*:ab,ti OR ischemi*:ab,ti OR ischaemi*:ab,ti OR infarct*:ab,ti OR haematoma*:ab,ti OR hematoma*:ab,ti OR bleed*:ab,ti))

Search strategy in Scopus July 1, 2020 (read from bottom-up).

Set	Search terms
#5	#4 AND (LIMIT-TO (SRCTYPE , "j")) AND (LIMIT-TO (DOCTYPE , "ar") OR LIMIT-TO (DOCTYPE , "re") OR LIMIT-TO (DOCTYPE , "ip")) AND (LIMIT-TO (LANGUAGE , "English"))
#4	#1 AND #2 AND #3
#3	(TITLE-ABS-KEY (movement OR motion OR mechanical OR biomechanic* OR kinematic* OR kinetic* OR angle* OR force* OR motion OR acceler* OR deceler* OR rotation OR velocity* OR speed* OR spatiotemporal))
#2	(TITLE-ABS-KEY (pronation OR supination OR hand AND strength OR reach* OR coordination OR grasp* OR grip* OR pinch AND strength OR "Upper Extremit*" OR "Upper Limb*" OR arm OR arms OR shoulder OR elbow* OR forearm* OR wrist* OR hand OR hands OR finger* OR thumb*))
#1	((TITLE-ABS-KEY (cva OR cvas OR poststroke* OR stroke* OR apoplex*) OR TITLE-ABS-KEY (((brain* OR cerebr* OR cerebell* OR intracran* OR intracerebral* OR vertebrobasilar*) AND vascular* AND (disease OR diseases OR accident* OR disorder*))) OR TITLE-ABS-KEY (((brain* OR cerebr* OR cerebell* OR intracran* OR intracerebral* OR vertebrobasilar*) AND (haemorrhag* OR hemorrhag* OR ischemi* OR ischaemi* OR infarct* OR haematoma* OR hematoma* OR bleed*)))))

Search strategy in the Cochrane Library July 1, 2020 (read from bottom-up).

Set	Search terms
#4	#1 AND #2 AND #3 in Cochrane Reviews (Reviews and Protocols), Other Reviews and Trials
#3	Movement or Motion or Mechanical or biomechanic* or Kinematic* or kinetic* or angle* or force* or motion or acceler* or deceler* or rotation or velocity* or speed* or spatiotemporal:ti,ab,kw
#2	Pronation or Supination or Hand Strength or reach* or coordination or grasp* or grip* or pinch strength or "Upper Extremit*" or "Upper Limb*" or arm or arms or shoulder or elbow* or forearm* or wrist* or hand or hands or finger* or thumb*:ti,ab,kw
#1	cva or cvas or poststroke* or stroke* or apoplex* or ((brain* or cerebr* or cerebell* or intracran* or intracerebral* or vertebrobasilar*) and vascular* and (disease or diseases or accident* or disorder*)) or (cerebrovascular* and (disease or diseases or accident* or disorder*)) or ((brain* or cerebr* or cerebell* or intracran* or intracerebral* or vertebrobasilar*) and (haemorrhag* or hemorrhag* or ischemi* or ischaemi* or infarct* or haematoma* or hematoma* or bleed*)):ti,ab,kw
