# Supplementary material for: Quantifying Quality of Reaching Movements Longitudinally Post-Stroke: A Systematic Review
Source: Neurorehabil Neural Repair. 2022 Jan 31;36(3):183–207. doi: 10.1177/15459683211062890 (PMC8902693; doi:10.1177/15459683211062890)
Supplement: sj-pdf-2-nnr-10.1177_15459683211062890 – Supplemental Material for Combined Quantifying Quality of Reaching Movements Longitudinally Post-Stroke: A Systematic Review [file sj-pdf-2-nnr-10.1177_15459683211062890.pdf]

Appendix B. Glossary: Definitions used in this study

Stroke: an episode of acute neurological dysfunction presumed to be caused by ischemia or hemorrhage, persisting ≥24 hours or until death.[1]

Motor control: the process by which motor commands produced by the CNS activate and coordinate muscles to generate joint torques to move effectors in goal-directed actions.[2]

Quality of Movement: patient's motor execution of a task in comparison with age-matched norm values of healthy individuals[3], closely related with the level of impairment.[4]

Recovery: reflects the extent to which body function and structure, as well as activities, have returned to their pre-stroke state[5].

Motor impairment: problems in body function and structure such as a significant deviation or loss related to movement[6].

Spontaneous neurological recovery: recovery over time irrespective of the type and amount of therapeutic interventions determined by certain unknown underlying biological processes.[7]

Behavioral restitution: a return towards more normative patterns of motor control with the impaired effector (a body part such as the hand or foot that interacts with an object or the environment) and reflects the process towards true recovery.[5]

Behavioral compensation: recovery of the ability to complete a motor task due to the appearance of new motor patterns resulting from the adaptation of remaining motor elements or substitution, meaning that functions are taken over, replaced, or substituted by different end effectors or body segments (Levin 2009).[4] Behavioural compensation results in deviating quality of movement compared to healthy individuals.

Reaching: active extension of the elbow and/or flexion of the shoulder and possibly flexion of trunk in order to either point to or grasp an object.

Performance assay: tests that quantify aspects of affected motor control deficits that underlie disruptions in functional movements more or less isolated from the context of the functional task.[3] These assays best capture the true upper bound of the degree of true neurological recovery[3] and are thereby not affected by compensation strategies.

Kinematic metric: a metric of body movement.

Kinetic metric: a metric of forces from or on body segments.

Responsiveness: sensitivity to change over time.

Longitudinal relation: association between covariates over time.

References

1. Sacco RL, Kasner SE, Broderick JP, et al. An updated definition of stroke for the 21st century: A statement for healthcare professionals from the American heart association/American stroke association. *Stroke*. 2013;44:2064–2089.

2. Haith AM, Krakauer JW. Theoretical Models of Motor Control and Motor Learning. In: *Routledge Handbook of Motor Control and Motor Learning*. Routledge; 2015:7–28.

3. Kwakkel G, Van Wegen E, Burridge JH, et al. Standardized measurement of quality of upper limb movement after stroke: Consensus-based core recommendations from the Second Stroke Recovery and Rehabilitation Roundtable. *Int. J. Stroke*. 2019;14:783–791.

4. Levin MF, Kleim JA, Wolf SL. What Do Motor "Recovery" and "Compensation" Mean in Patients Following Stroke? *Neurorehabil. Neural Repair*. 2009;23:313–319.

5. Bernhardt J, Hayward KS, Kwakkel G, et al. Agreed Definitions and a Shared Vision for New Standards in Stroke Recovery Research: The Stroke Recovery and Rehabilitation Roundtable Taskforce. *Neurorehabil. Neural Repair*. 2017;31:793–799.

6. WHO. *International classification of functioning, disability and health. Geneva:*; 2001.

7. Kwakkel G, Kollen B, Twisk J. Impact of time on improvement of outcome after stroke. *Stroke*. 2006;37:2348–2353.
