# Supplementary material for: Quantifying Quality of Reaching Movements Longitudinally Post-Stroke: A Systematic Review
Source: Neurorehabil Neural Repair. 2022 Jan 31;36(3):183–207. doi: 10.1177/15459683211062890 (PMC8902693; doi:10.1177/15459683211062890)
Supplement: sj-pdf-3-nnr-10.1177_15459683211062890 – Supplemental Material for Combined Quantifying Quality of Reaching Movements Longitudinally Post-Stroke: A Systematic Review [file sj-pdf-3-nnr-10.1177_15459683211062890.pdf]

APPENDIX C.

Checklist for stroke recovery and rehabilitation studies measuring kinematics and kinetics of the upper extremity.

✓	Item	Source*
	Did the longitudinal repeated measures study at least meet the criteria of the STROBE statement for cohort studies?	STROBE
	Did the study describe at least the following set of subject characteristics?<br>Age, gender, arm dominance, severity of stroke within 3 days after stroke onset (NIHSS), stroke type (ischemic / haemorrhagic), stroke sub-type (lacunar / large artery / other / undetermined), stroke location (cortical / subcortical / midbrain / brainstem), thrombolysis / reperfusion therapy (yes / no).	SRRR1
	Did the study describe the presence of active hand movement (yes/no) at stroke onset?	SRRR1
	Did the study perform measurements at fixed moments after stroke onset?	SRRR2
	Did the study obey the recommended timing of assessments early post stroke? (at week 1, week 12, and week 26 after stroke onset)	SRRR2
	Did the study include the recommended clinical assessment at the level of Body function and structure? (FM-UE)	SRRR1
	Did the study include the recommended clinical assessment at the level of Activity?(ARAT)	SRRR1
	Did the study include measuring any of the recommended kinetic and kinematic performance assays (respectively, pinch and grasp strength, and elbow extension and finger individuation)?	SRRR2
	Did the study include a functional drinking task, or an alternative standardized reaching task?	SRRR2; (Mesquita et al., 2020)
	Did the study perform measurements with high speed or high resolution measurement equipment?	SRRR2
	Did the study satisfy the recommended minimal sample frequency of 60Hz?	SRRR2
	Did the study satisfy the recommended minimal number of repetitions for each movement? (n=15)	SRRR2
	Did the study include or refer to available data of an age-matched healthy control group?	SRRR2
	Did the study analyze the longitudinal change in the kinematics/kinetics over time?	SRRR2
	Did the study encompass FAIR data presentation (Findable, Accessible, Interoperable, Reusable)?	FAIR

*STROBE: cohort checklist, https://www.strobe-statement.org/index.php?id=available-checklists; SRRR1: Kwakkel et al., 2017 (DOI: 10.1177/1747493017711813); SRRR2: Kwakkel et al., 2019 (DOI: 10.1177/1545968319886477); FAIR: Wilkinson et al., 2016 (DOI: 10.1038/sdata.2016.18.). *Abbreviations:* ARAT: Action Research Arm Test; FM-UE: Fugl-Meyer motor assessment of the upper extremity; mRS: modified Rankin Scale; NIHSS: National Institutes of Health Stroke Scale; SRRR: Stroke Recovery and Rehabilitation Roundtable task force.
