# Supplementary figures and images for: Quantifying Quality of Reaching Movements Longitudinally Post-Stroke: A Systematic Review
Source: Neurorehabil Neural Repair. 2022 Jan 31;36(3):183–207. doi: 10.1177/15459683211062890 (PMC8902693; doi:10.1177/15459683211062890)

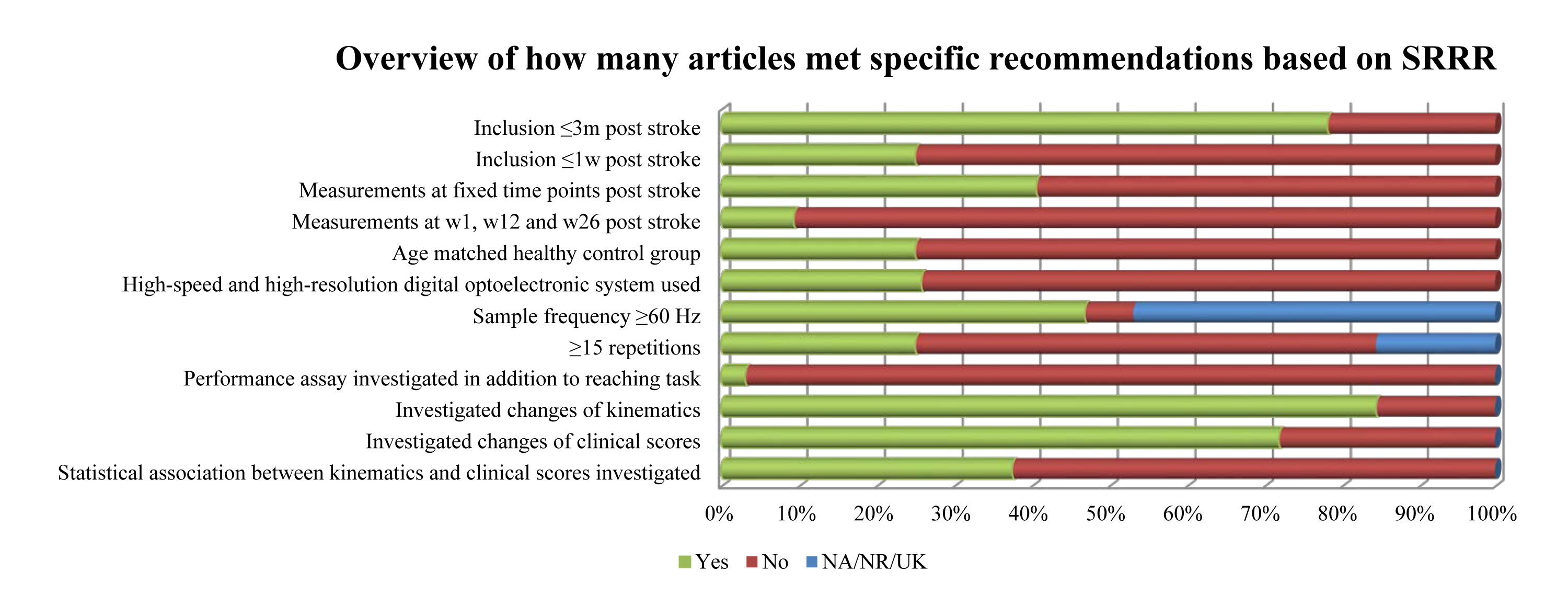

Supplement: sj-jpg-4-nnr-10.1177_15459683211062890 – Supplemental Material for Combined Quantifying Quality of Reaching Movements Longitudinally Post-Stroke: A Systematic Review [file sj-jpg-4-nnr-10.1177_15459683211062890.jpg]
