# Supplementary material for: Quantifying Quality of Reaching Movements Longitudinally Post-Stroke: A Systematic Review
Source: Neurorehabil Neural Repair. 2022 Jan 31;36(3):183–207. doi: 10.1177/15459683211062890 (PMC8902693; doi:10.1177/15459683211062890)
Supplement: sj-pdf-5-nnr-10.1177_15459683211062890 – Supplemental Material for Combined Quantifying Quality of Reaching Movements Longitudinally Post-Stroke: A Systematic Review [file sj-pdf-5-nnr-10.1177_15459683211062890.pdf]

Table 3. Overview of whether studies are in agreement with the recent recommendations of SRRR

	Measurement time points				Measurement methods				
	Inclusion in or before early sub-acute phase (≤3 months) post stroke	*Inception cohort from stroke onset onwards (≤1week)*	*Measurements at fixed time points post stroke*	*Measurement moments at least at: w1, w12 and w26*	*Age matched healthy control group*	*High speed and high resolution digital optoelectronic system used*	*Sample frequency >60Hz*	*>15 repetitions per task*	*In addition, a performance assay was investigated to identify behavioral restitution*
**Platz et al., 2001**	1	0	0	0	0	0	1	1	0
**Rohrer et al., 2002**	1	0	0	0	0	0	NR	NR	0
**Lang et al., 2006a**	1	1	1	0	0	1	1	0	0
**Lang et al., 2006b**	1	1	1	0	1	1	1	0	0
**Wagner et al., 2007**	1	1	1	0	1	1	1	0	1
**Konczak et al., 2010**	1	0	0	0	1	0	1	0	0
**Edwards et al., 2012**	1	1	1	0	0	1	1	0	0
**Tan et al., 2012**	0	0	0	0	1	0	NA	0	0
**Dipietro et al., 2012**	1	0	0	0	0	0	NR	1	0
**Van Kordelaar et al., 2013**	1	0	1	0	1	0	1	0	0
**Colombo et al., 2013**	0	0	0	0	0	0	1	NR	0
**Duret and Hutin 2013**	1	0	0	0	0	NR	NR	0	0
**Metrot et al., 2013a**	1	0	0	0	0	0	0	0	0
**Van Kordelaar et al., 2014**	1	1	1	1	0	0	1	0	0
**Van Dokkum et al., 2014**	1	0	0	0	0	0	0	0	0
**Krebs et al., 2014**	1	1	1	0	0	0	NR	UK	0
**Yoo et al., 2015**	0	0	0	0	0	0	NR	NR	0
**Semrau et al., 2015**	1	1	1	1	0	0	NR	NR	0
**Li et al., 2015**	0	0	0	0	0	1	1	0	0
**Bang et al., 2015**	0	0	0	0	0	1	NR	0	0
**Prange et al., 2015**	1	0	0	0	0	0	NR	0	0
**Buma et al., 2016**	1	0	1	0	0	0	1	0	0
**Duret et al., 2016**	0	0	0	0	0	0	NR	1	0
**Cortes et al., 2017**	1	0	1	0	1	0	1	1	0
**Pila et al., 2017**	1	0	1	0	1	0	NR	1	0
**Palermo et al., 2018**	0	0	0	0	0	1	1	0	0
**Mazzoleni et al., 2018**	1	0	0	0	0	0	NR	0	0
**Duret et al., 2019**	1	0	0	0	0	0	NR	1	0
**Mazzoleni et al., 2019**	1	0	0	0	0	0	NR	0	0
**Goffredo et al., 2019**	1	0	0	0	0	0	1	1	0
**Hussain et al., 2020**	1	0	1	0	0	0	NR	1	0
**Thrane et al., 2020**	1	1	1	1	1	1	1	0	0
**TOTAL (yes/total)**	**25/32**	**8/32**	**13/32**	**3/32**	**8/32**	**8/32 (1NR)**	**15/17 (14NR, 1NA)**	**8/27 (4NR, 1UK)**	**1/32**

Abbreviations: 0, did not meet criterion; 1, did meet criterion; NA, Not applicable; NR, Not reported; UK, unknown/unclear; w, weeks post stroke.
